# Leakage-free evaluation of task-aware Nyström sampling for IoMT intrusion detection

**DOI:** 10.3389/frai.2026.1957727

**Published:** 2026-09-28

**Authors:** Hiba A. Tarish, Rosilah Hassan, Khairul Akram Zainol Ariffin, Mustafa Musa Jaber

**Affiliations:** 1 Faculty of Information Science and Technology (FTSM), Centre for Cyber Security, Universiti Kebangsaan Malaysia (UKM), Bangi, Selangor, Malaysia; 2 Department of Civil Engineering, University of Technology, Baghdad, Iraq; 3 Informatics Institute for Postgraduate Studies, University of Information Technology and Communications, Baghdad, Iraq; 4 Department of Cyber Security, Al-Hikma University College, Baghdad, Iraq

**Keywords:** anomaly detection, data leakage, imbalanced classification, importance sampling, Internet of Medical Things, intrusion detection, landmark selection, Nyström kernel approximation

## Abstract

Standard Nyström kernel approximation samples landmark points uniformly at random, ignoring that boundary-adjacent and minority-class samples often matter most for imbalanced intrusion detection. We propose a task-aware, importance-weighted Nyström landmark-selection mechanism that scores each training sample's classifier uncertainty, boundary distance, representativeness, and minority-class importance, with an optional quantum-amplitude reparameterization (QI-AWNS) shown to be algebraically equivalent to direct score-proportional sampling; the contribution is the importance scoring and its rigorous evaluation, not the amplitude framing or a quantum advantage. We benchmark QI-AWNS against uniform, *K*-means, and approximate ridge-leverage-score Nyström sampling, five tabular baselines, and a full-kernel RBF-SVM on two leakage-free IoMT benchmarks (ECU-IoHT, WUSTL-EHMS-2020) under a strict split-before-fit protocol, with each test partition evaluated once. Across 10 reseeded splits, per-split retuning of the importance weights improves QI-AWNS's stability relative to a frozen-weight configuration and yields a mean balanced-accuracy gain of 0.18 points over uniform sampling on ECU-IoHT. This is not significant after Holm correction for multiple comparisons (raw Wilcoxon *p* = 0.022, adjusted *p* = 0.129), and no advantage appears over *K*-means or approximate ridge-leverage-score sampling on either dataset. An 8-variant ablation and weight-search analysis shows that a validation-based ranking of two weight candidates reverses on WUSTL-EHMS-2020's test set. These findings show that task-aware landmark weighting offers limited, dataset-dependent benefit, and that single-split conclusions can overstate it.

## 1 Introduction

The Internet of Medical Things (IoMT), comprising wearable biosensors, implantable devices, and hospital gateway systems that continuously collect, transmit, and analyze patient telemetry, has become integral to modern healthcare delivery, extending continuous monitoring beyond the hospital and easing the load on intensive-care and telemedicine infrastructure (Shi et al., 2016). This benefit comes with a cost: IoMT devices are safety-critical, resource-constrained, and frequently deployed on flat, weakly defended networks, making them attractive targets for cyberattacks whose consequences extend beyond data confidentiality to patient safety itself, since falsified vital-sign readings or disrupted monitoring streams can directly endanger a patient's life (Hady et al., 2020; Ahmed et al., 2021). The broader IoMT network-security landscape, including its resources, ecosystem, and interoperability challenges, along with the surrounding threat-mitigation literature, has been comprehensively surveyed elsewhere (Tarish et al., 2024). Machine-learning-based cyber threat intelligence has likewise been proposed for non-independent-and-identically-distributed data in related software-defined-networking settings (Kazmi et al., 2025). This study instead focuses on a narrower, complementary problem: leakage-free, lightweight intrusion detection, addressed through a split-before-fit protocol, matched-budget baselines, and statistically corrected significance testing (Sections 3.2–3.10). We do not evaluate adversarial robustness, explainability, fairness, or accountability, which we explicitly return to in Section 6.

Machine learning intrusion detection systems (IDS) are widely proposed to meet this need, and much of that literature reports accuracies at or above 99%. Two problems complicate taking these figures at face value. First, a substantial fraction of this literature is vulnerable to *data leakage*, in which information from the test partition influences feature selection, scaling, or hyperparameter search, inflating reported accuracy in ways that do not generalize to deployment; Kapoor and Narayanan documented this failure mode across 17 scientific fields (Kapoor and Narayanan, 2023), and Bouke and Abdullah showed it concretely for network intrusion detection (Bouke and Abdullah, 2023). Second, many of the most accurate reported models are deep or ensemble architectures whose computational cost (memory footprint, training time, and inference latency) is difficult to reconcile with the low-power gateways and embedded microcontrollers that constitute much of the IoMT edge (Shi et al., 2016).

Lightweight kernel-approximation methods offer a candidate solution to the second problem. Constructing a full kernel machine requires computing and storing the entire *N* times *N* kernel, or Gram, matrix, which consumes *O*(*N*^2^) memory. Downstream operations such as matrix inversion or eigendecomposition on this matrix can then scale superlinearly, or even cubically, in the number of samples *N*, a cost that is prohibitive for the large-scale or resource-constrained deployments targeted in this paper. Random-feature and Nyström approximations instead project the input into a finite-dimensional randomized or landmark-based feature space, where a simple linear classifier can approximate the same decision boundary a full kernel machine would find, at a fraction of the cost (Rahimi and Recht, 2007; Williams and Seeger, 2001). The standard Nyström method selects its landmark points *uniformly at random*, treating every training sample as equally informative; this ignores that boundary-adjacent, locally rare, and minority-class samples frequently most determine a decision boundary in an imbalanced intrusion-detection setting. *K*-means-based landmark selection (Zhang et al., 2008) and approximate ridge-leverage-score sampling (Alaoui and Mahoney, 2015) were proposed in the classical kernel-approximation literature as alternatives that weight landmark selection by data geometry rather than sampling uniformly; we adopt both as comparison baselines in this study (Section 3.6).

This construction also shares mathematical lineage with the classical, “dequantized” algorithms that emerged from Tang's result, which showed that several proposed quantum machine-learning speedups can be matched, up to polynomial overhead, by classical algorithms with equivalent data access (Tang, 2019; Huynh et al., 2023); we discuss this connection in Section 2. Building on this, we name our four-component importance-weighted landmark-selection mechanism QI-AWNS (Quantum-Inspired Amplitude-Weighted Nyström Sampling, Section 3.6): each candidate landmark's combined importance score can optionally be re-expressed as a normalized quantum-style amplitude and sampled with probability equal to the squared amplitude, a Born-rule-style reparameterization of the same importance-weighted sampling step. This reparameterization is not a core methodological feature or a source of novelty. We state plainly that QI-AWNS involves no quantum hardware and no computational quantum advantage; Section 3.6 proves algebraically that the amplitude construction is exactly equivalent, for every input, to direct score-proportional normalization, contributing no numerical difference whatsoever. Readers can read every subsequent use of “QI-AWNS” simply as “the four-component importance-weighted sampler” with no loss of technical content; this study's actual contribution is the four-component importance scoring and its rigorous evaluation against strong classical alternatives.

This study contributes a task-aware landmark-selection framework, evaluated honestly (including cases where the result is narrow, mixed, or negative) against strong classical alternatives under a leakage-free protocol, with an optional quantum-amplitude reparameterization of the sampling step that is disclosed but not load-bearing. Concretely, this research makes the following contributions:

1. We propose a task-aware, importance-weighted Nyström landmark-selection mechanism that combines classifier uncertainty, boundary distance, local representativeness, and minority-class importance into a single score. Section 3.6 shows that this score admits an optional quantum-amplitude reparameterization (QI-AWNS) that is mathematically equivalent to direct score-proportional sampling; the importance-weighting mechanism, not the amplitude framing, is the primary contribution.
2. We design, document, and code-verify a leakage-free evaluation pipeline: within each evaluation split (Section 3.2 details the single-split protocol; Section 3.10 the 10 reseeded splits used for the landmark-selection comparison), a stratified 70/15/15 train/validation/test partition is created *before* feature selection, scaling, or landmark/hyperparameter search; feature selectors and scalers are fit only on the training partition. All search and importance-weight tuning is scored exclusively on the validation partition, and the test partition is evaluated exactly once, at the end.
3. We benchmark QI-AWNS against uniform, *K*-means (Zhang et al., 2008), and approximate ridge-leverage-score (Alaoui and Mahoney, 2015) Nyström landmark selection under matched kernel hyperparameters and a shared classifier, isolating landmark-selection strategy as the sole varying factor. We evaluate across 10 reseeded train/validation/test splits under two distinct protocols (frozen importance weights vs. independently retuned per seed) and use McNemar's exact test (single split) and Wilcoxon signed-rank tests (across reseeds, both protocols) for pairwise significance.
4. We run an 8-variant ablation over QI-AWNS's four scoring components (each in isolation, the full combination, a deterministic top-*m* variant that removes probabilistic sampling, and an equal-weighted variant), and a small validation-only grid search over the combination weights, and report a validation-tuning overfitting effect that this search exposes on WUSTL-EHMS-2020.
5. We report accuracy, balanced accuracy, precision, recall, F1-score, specificity, Matthews correlation coefficient, ROC-AUC, PR-AUC, Wilson-score confidence intervals (Wilson, 1927), fit time, and per-sample inference latency for each method compared, and candidly discuss why the resulting leakage-free numbers are markedly lower than the accuracies above 99% reported in prior literature for these datasets.

The remainder of this paper is organized as follows. Section 2 reviews related work, organized by dataset, covering IoHT/IoMT intrusion detection, the quantum-inspired/dequantized machine-learning literature, and classical landmark-selection strategies for the Nyström method. Section 3 describes the datasets, the leakage-free pipeline, the full mathematical construction of QI-AWNS and the comparison landmark strategies, and the linear classification stage. Section 4 reports experimental results: the main landmark-selection comparison, the ablation and weight-search findings, and the tabular baselines. Section 5 discusses the cross-method and cross-dataset findings, the gap relative to prior literature, and practical deployment implications. Section 6 states limitations. Section 7 concludes with future work.

## 2 Related work

### 2.1 Quantum-inspired and kernel-approximation machine learning

Genuine quantum machine learning remains constrained by hardware noise and limited qubit counts, motivating classical approximations that borrow the geometric intuition of high-dimensional Hilbert-space feature maps without requiring quantum hardware (Huynh et al., 2023). Tang's dequantization result showed that several proposed quantum recommendation-system speedups can be matched, up to polynomial overhead, by classical sampling-based algorithms with equivalent data access (Tang, 2019), thereby motivating a broader family of “quantum-inspired” classical methods.

Chia et al. (2022) subsequently generalized this line of work into a unified sampling-based framework that encompasses essentially all known quantum linear-algebra techniques proposed for machine learning, offering more recent, broader evidence that this dequantization phenomenon is not limited to Tang's original recommendation-system setting.

Independently of this quantum connection, random-feature and Nyström kernel approximations were developed within classical kernel-methods research to make large-scale kernel machines computationally tractable: Rahimi and Recht's random Fourier features approximate a shift-invariant kernel via randomized projections (Rahimi and Recht, 2007), and Williams and Seeger's Nyström method approximates a kernel matrix from a small set of landmark points (Williams and Seeger, 2001). Both constructions predate and are mathematically independent of the quantum-inspired label; we use them here as classical kernel-approximation tools in their own right, discussing the quantum-inspired framing only as context for QI-AWNS's amplitude-and-Born-rule-style sampling interpretation (Section 3.6), not as a claim about the underlying method's origin or mechanism.

### 2.2 Landmark selection for the Nyström method

Because Nyström approximation quality depends on how well the chosen landmarks represent the full kernel matrix's spectrum, a body of classical work replaces uniform random landmark sampling with data-dependent alternatives. Zhang et al. (2008) proposed selecting landmarks as *K*-means cluster centroids rather than raw data points, showing that this improves low-rank approximation error relative to uniform sampling. Alaoui and Mahoney (2015) proposed ridge-leverage-score sampling, in which the landmark-inclusion probability is proportional to each point's statistical leverage under a ridge-regularized kernel, providing approximation-quality guarantees that uniform sampling lacks.

This builds on Drineas and Mahoney (2005)'s earlier foundational analysis, which establishes formal error bounds for Gram-matrix Nyström approximation under both uniform and non-uniform column-sampling schemes.

Both methods weight landmark selection based on the data's geometric properties; QI-AWNS (Section 3.6) instead weights landmark selection using a task-specific combination of classifier uncertainty, decision-boundary proximity, local density, and class-imbalance-aware importance. We compare directly with *K*-means and approximate ridge-leverage-score landmark selection in Section 4, rather than only with uniform sampling, because a fair test of QI-AWNS's value must include the existing non-uniform alternatives.

### 2.3 ECU-IoHT related work

Ahmed et al. (2021) introduced ECU-IoHT, an IoHT testbed that exercises reconnaissance, man-in-the-middle, and denial-of-service attacks against medical devices. Table 1 summarizes the accuracy, model class, and split-before-fit documentation status of the studies that followed; a few qualitative findings beyond the tabulated numbers are worth noting. Vijayakumar et al. (2023)'s 99.85% deep-neural-network accuracy conceals a recall of only approximately 60% for infrequent denial-of-service attacks, illustrating how a high headline accuracy can mask weak minority-class detection; Algethami and Alshamrani (2024)'s comparable-accuracy hybrid framework similarly shows lower precision on Smurf-type attacks. Areia et al. (2024) found naïve Bayes markedly less stable than random forest and decision tree among six benchmarked classifiers, and Alohali et al. (2025)'s EIoHTSCD-SEGO framework was evaluated only on a static dataset. Mosaiyebzadeh et al. (2025)'s federated-learning CNN trades some accuracy for stronger privacy guarantees. Most recently, Zhao (2025)'s hybrid Decision-Tree/Deep-Q-Network framework was evaluated for cross-dataset generalization on WUSTL-EHMS, ECU-IoHT, DF_IOMT, and CICIOT23, one of the few studies evaluating a single model across both primary benchmarks used here, again without a documented split-before-fit protocol.

**Table 1:** Related-work survey, Part A (ECU-IoHT): dataset, documented split-before-fit protocol, model class, reported accuracy, and relative model complexity.

Paper	Dataset	Split-before-fit protocol documented?	Model	Acc. (%)	Relative complexity
Vijayakumar et al. (2023)	ECU-IoHT	Not documented	Deep neural network	99.85	High (deep net)
Areia et al. (2024)	ECU-IoHT	Not documented	RF/DT/NB/LR/SVM/DNN (best: RF/DT)	>99 F1	Low–High (mixed)
Algethami and Alshamrani (2024)	ECU-IoHT	Not documented	Hybrid deep-learning framework	99.85	High (hybrid DL)
Alohali et al. (2025)	ECU-IoHT	Not documented	EIoHTSCD-SEGO (optimization + ensemble)	99.33	High (multi-stage)
Mosaiyebzadeh et al. (2025)	ECU-IoHT, WUSTL	Not documented	Federated-learning CNN	95.48 / 93.20	Moderate–High (federated)
Alharbi and Khan (2025)	ECU-IoHT	Not documented	Random forest (best of 5)	98.00	Low (tree ensemble)
Krishna Kumari et al. (2026)	ECU-IoHT, WUSTL	Not documented	MREQGAN-DSA (quantum-driven, multi-component)	99.81 / 99.81	High (multi-component optimization)

Caveat: our ECU-IoHT results use the 4-raw-feature version of this dataset available to us (source, destination, protocol, length; Section 3.1), which is substantially narrower than the richer feature sets (traffic statistics, time-window features) reported in some of the studies above. The accuracy gap against these studies is therefore consistent with at least two distinct, non-exclusive explanations—our leakage-free protocol, and/or this feature-count difference—and we cannot attribute it to either specifically without reproducing the cited studies (Section 5, Section 6).

Taken together, ECU-IoHT studies frequently exceed 99% accuracy, but we did not identify any of the studies reviewed above that report a leakage-free split-before-fit protocol, a matched hyperparameter-search budget across the compared models, or nested cross-validation with per-fold preprocessing refitting. This study addresses these gaps with the lightweight model evaluated here.

### 2.4 WUSTL-EHMS-2020 related work

Hady et al. (2020) introduced WUSTL-EHMS-2020, an Enhanced Healthcare Monitoring System testbed that combines patient biometrics with network flow metrics and shows that combining both feature families outperforms either alone. Table 2 summarizes the accuracy, model class, and split-before-fit documentation status of the studies that followed. Beyond the tabulated figures, several qualitative findings stand out: Judith et al. (2023)'s PCA-plus-multilayer-perceptron approach shows a comparatively high false-positive rate in overlapping multiclass settings despite its 96.39% accuracy; Shaikh et al. (2024)'s RCLNet was evaluated only on historical, non-streaming data; Wu et al. (2025)'s stacked deep-learning framework underperforms RCLNet despite greater architectural complexity; and Aversano et al. (2026)'s SurIoT, despite reaching approximately 99.85% accuracy in some settings, has its F1-score fall to 83.08% for slow-rate denial-of-service attacks, attributed to its stateless, axis-aligned rules. As for ECU-IoHT, we did not find a WUSTL-EHMS-2020 study documenting a split-before-fit protocol at the level of detail Section 3.2 provides; we return to this gap in Section 5 when we report our own, markedly lower, leakage-free numbers.

**Table 2:** Related-work survey, part B (WUSTL-EHMS-2020 and this work).

Paper	Dataset	Split-before-fit protocol documented?	Model	Acc. (%)	Relative complexity
Tauqeer et al. (2022)	WUSTL	Not documented	RF / gradient boosting / SVM	96.90 / 96.50 / 95.85	Low (conventional ML)
Judith et al. (2023)	WUSTL	Not documented	PCA + multilayer perceptron	96.39	Moderate
Shaikh et al. (2024)	WUSTL	Not documented	RCLNet (CNN + LSTM)	99.78	High (deep hybrid)
Wu et al. (2025)	WUSTL	Not documented	Stacked deep-learning ensemble	94.29	High (stacked DL)
Balhareth et al. (2026)	WUSTL	Not documented	ML-FSID-FIS (fuzzy inference)	~93	Moderate
Abid (2026)	WUSTL	Not documented	Trust-based hard-voting ensemble	95.00	Moderate (ensemble)
Aversano et al. (2026)	WUSTL	Not documented	SurIoT (stateless, axis-aligned rules)	~99.85	Low (rule-based)
Abdelhaq et al. (2026)	WUSTL	Not documented	XGBoost + PCA + SVM	98.04	Moderate
This work	ECU-IoHT, WUSTL	Yes (Section 3.2)	Nyström/random-feature (RFK) + QI-AWNS, *m* = 100–600	96.39 / 92.89	Low (linear classifier on *m*-dim. embedding)

Two recent strands bear on this paper's framing of deployability and interpretability. A broad PRISMA-ScR review of 78 lightweight IoT/edge intrusion-detection studies found architectural compactness commonly reported, but target-device latency, runtime memory, and power inconsistently documented (Hossain, 2026). This gap is addressed in Section 3.6.1 and Table 3 for our own model, and we explicitly note that our measurements are likewise sandbox-CPU, not target-device (Section 6). On the interpretability side, Hosain and Çakmak (2025) combined SHAP- and LIME-based explainability with XGBoost for IoMT intrusion detection. This expectation is engaged via QI-AWNS's individually named, inspectable importance components (Section 5), rather than a *post-hoc* explainability layer.

**Table 3:** Landmark-selection efficiency (seed 42, Sandbox CPU, not edge hardware).

Dataset	Method	Fit (s)	Latency (ms)	Thr. (/s)	Size (KB)	RAM (MB)
ECU	Uniform	0.58	0.00023	4.35M	80.8	118.9
	*K*-means	0.80	0.00005	18.9M	80.8	119.1
	Approx. RLS	0.54	0.00003	39.7M	80.8	118.9
	QI-AWNS	0.99	0.00032	3.11M	80.8	118.9
*WUSTL	Uniform	2.56	0.00238	420K	2901.9	107.4
	*K*-means	2.31	0.00016	6.31M	2901.9	107.4
	Approx. RLS	1.89	0.00059	1.70M	2901.9	107.4
	QI-AWNS	2.29	0.00045	2.22M	2901.9	107.4

### 2.5 Summary of gaps

Across the two benchmark datasets, four recurring gaps motivate the present study, based on the studies discussed in Sections 2.3–2.4 (a narrative, per-dataset review rather than a formal systematic search with pre-declared inclusion criteria, Section 6). First, we did not identify any study that documents a leakage-free, split-before-fit pipeline in sufficient detail to rule out test-set influence on feature selection or scaling. Second, high aggregate accuracy often conceals weak minority-class detection, as in Vijayakumar et al. (2023), Algethami and Alshamrani (2024), Abid (2026), and Aversano et al. (2026). Third, the strongest-performing models are often deep, ensemble, or multi-component optimization architectures, and their computational cost is rarely reported on a comparable basis. Fourth, we did not identify any study that benchmarks a Nyström/random-feature lightweight model against both simple tabular baselines and a validation-only hyperparameter search matched in effort to a more complex competing architecture. This study addresses all four gaps for the lightweight model under study, while being explicit that doing so yields headline accuracy numbers lower than much of the cited prior literature (Section 5).

Tables 1, 2 consolidate the related-work studies discussed above into a single comparison (split across two tables for page layout). “Split-Before-Fit Protocol Documented?” indicates whether the cited study's published description documents a split-before-fit procedure comparable to Section 3.2; “Not documented” does not mean the study's results are leaked, only that its published description does not allow us to verify either way (Section 5). These reported results cannot be compared directly because previous studies did not use a verified split-before-fit evaluation protocol, which is precisely why that column is included here.

## 3 Materials and methods

### 3.1 Datasets

ECU-IoHT (Ahmed et al., 2021) was collected from an IoHT testbed at Edith Cowan University that conducted cyberattacks (including man-in-the-middle and reconnaissance activity) against simulated medical devices; the file available to us exposes four raw columns (source, destination, protocol, length), substantially fewer than the original publication's broader feature set, so our ECU-IoHT results pertain specifically to this feature-limited subset. WUSTL-EHMS-2020 (Hady et al., 2020) was collected from an Enhanced Healthcare Monitoring System testbed and combines patient biometric readings (heart rate, SpO2, pulse rate) with network flow metrics under spoofing and data-injection attacks; 18 of 37 raw features passed our training-set-only feature selection (Section 3.3). Class balance differs substantially: ECU-IoHT is attack-majority in our test partition, while WUSTL-EHMS-2020 is normal-majority, with the attack class comprising 12.5% of the test partition (307 of 2,448 samples), a class-imbalance regime with well-documented effects on both training and evaluation (He and Garcia, 2009). Our main results throughout the paper rely on training-derived class weighting rather than resampling (Section 3.7, Section 3.9); Section 4.2 additionally reports a direct comparison against SMOTE (Chawla et al., 2002) oversampling on WUSTL-EHMS-2020. All reported precision, recall, and F1-score values are for the attack (positive) class specifically.

### 3.2 Leakage-free evaluation pipeline

Undetected data leakage is, in our assessment, the single most common source of inflated accuracy claims in this literature (Kapoor and Narayanan, 2023; Bouke and Abdullah, 2023). All code implementing the protocol below, the landmark-selection methods, and the statistical tests was developed with the assistance of a generative AI tool under the corresponding author's direction and specification (see the Acknowledgments section for the full disclosure); every experiment was executed, and every reported value was verified against the underlying result files, by the corresponding author. For both primary datasets, we fix and verify in code, before running any experiment, the following protocol:

1. **Split before anything else**. Each dataset is split into training (70%), validation (15%), and test (15%) partitions using a single stratified split (random_state=42) applied to the raw feature matrix before any feature selection or scaling is fit. For the primary evaluation, the split uses random state 42. For the repeated landmark-selection analysis (Section 4.3), the same split-before-fit procedure is repeated independently for seeds 42 and 1–9.
2. **Feature selection fit on training data only**. A univariate selector (SelectPercentile, scored with the ANOVA F-statistic at the 50th percentile) is fit only on the training partition, then applied unchanged to validation and test.
3. **Scaling fit on training data only**. Z-score standardization (Equation 2, Section 3.4) is fit only on the training partition.
4. **Hyperparameter search scored on validation only**. The matched-budget search described in Section 3.8 trains and scores each candidate only on the training and validation partitions; the test partition is never passed to the search.
5. **Single held-out test evaluation**. After hyperparameter selection and final refitting, the model is evaluated exactly once on the untouched test partition. All accuracy, precision/recall/F1, and ROC-AUC results reported in Section 4 for the primary evaluation are from that single evaluation.
6. **Cross-validation for variance estimation**. A stratified 5-fold cross-validation is run on the pooled training+validation data with feature selection and scaling refit fresh *inside every fold* (via a single scikit-learnPipeline wrapping selector, scaler, feature map, and classifier), avoiding the optimistic bias that results from fitting these steps once outside the fold loop, a bias present, by our reading, in several prior studies' reported cross-validation figures for this class of model.
7. **Landmark selection and importance-weight tuning scored on validation only**. The four landmark-selection strategies compared in Section 3.6 (uniform, *K*-means, approximate ridge-leverage-score, QI-AWNS) are evaluated under matched kernel hyperparameters (Section 3.6); QI-AWNS's own importance-score combination weights are selected via a small grid search scored only on validation balanced accuracy (Section 4.3), never on the test partition.

### 3.3 Statistical feature selection: ANOVA F-Classif

This step is executed first in the pipeline (Section 3.2): a supervised, filter-based feature-selection step ranks features by the ANOVA F-statistic, computed as


$$\begin{array}{c} F=\frac{\text{MSB}}{\text{MSW}}=\frac{\text{SSB}/\left(K-1\right)}{\text{SSW}/\left(N-K\right)}, \end{array}$$ (1)

where SSB (between-groups sum of squares) measures how far each class's feature mean deviates from the overall mean, SSW (within-groups sum of squares) measures internal variance within each class, *K* is the number of target classes, and *N* is the number of training samples. A large *F*-statistic indicates that a feature separates the classes more than would be expected from within-class noise alone. Features are ranked in descending order of *F*, and the top 50th percentile is retained (SelectPercentile), fit on the training partition only per Section 3.2. On WUSTL-EHMS-2020, 7 of the 37 raw features are constant (zero-variance) within the training partition, producing an undefined (0/0) *F*-statistic for those columns. We treat these as *F* = 0 (no discriminative information) for ranking purposes, rather than allow an undefined value to be mishandled by a naive descending sort, and report this explicitly (Figure 1) rather than silently drop or ignore the affected columns.

**Figure 1:**
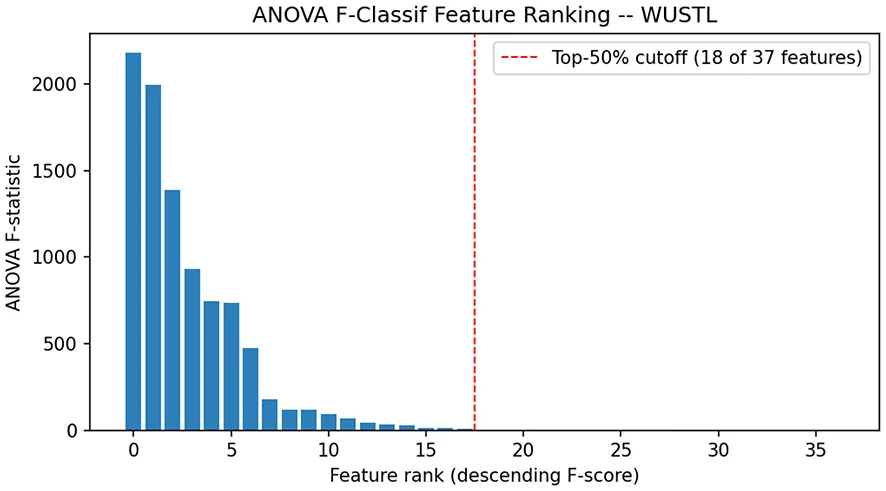
ANOVA F-statistic feature ranking, WUSTL-EHMS-2020 (37 raw features, 7 constants in the training partition and treated as *F* = 0; top-50% selection cutoff marked).

### 3.4 Data standardization

Standardization is applied second in the pipeline (Section 3.2), fit to the training partition's already-selected columns (Section 3.3) rather than to the raw feature matrix; this ordering has no effect on which features are selected, since the ANOVA *F*-statistic (Equation 1) is invariant to per-feature location-scale transformations (scaling or shifting a single feature multiplies both its between- and within-groups sum of squares by the same constant, leaving their ratio unchanged), but it does matter for the kernel mapping described below. Each selected feature is standardized via Z-score normalization,


$$\begin{array}{c} z_{i}=\frac{x_{i}-\mu }{\sigma }, \end{array}$$ (2)

where μ and σ are the training-partition mean and standard deviation for that feature, computed after selection. This step matters specifically because the RBF-kernel-based feature maps used in Section 3.5 are distance-sensitive: without isotropic per-feature variance, features with larger raw magnitude would dominate the kernel's implicit distance computation regardless of their actual discriminative value.

### 3.5 Random-feature and Nyström kernel mapping

A full kernel machine requires constructing and storing the *N*×*N* Gram matrix *K*_*ij*_ = *k*(*x*_*i*_, *x*_*j*_), which consumes *O*(*N*^2^) memory; downstream operations such as matrix inversion or eigende composition on this matrix are solver-dependent but can scale superlinearly or even cubically in *N*, which is prohibitive for the sample sizes and low-power hardware this paper targets. Random-feature and Nyström approximations instead construct an explicit, finite-dimensional feature map ϕ:ℝ^*d*^ → ℝ^*m*^ such that ϕ(*x*)^⊤^ϕ(*y*)≈*k*(*x, y*), so that a linear classifier trained on ϕ(*x*) approximates the decision boundary a full kernel machine would find, at a cost linear rather than cubic in sample count.

For ECU-IoHT, we use an RBFSampler (Rahimi and Recht, 2007's random Fourier features), which approximates the RBF kernel via (Equation 3).


$$\begin{array}{c} \phi \left(x\right)=\sqrt{\frac{2}{m}}\cos\left(\omega^{⊤}x+b\right), \end{array}$$ (3)

with ω~N(0,2γI), *b*~Unif(0, 2π), and *m* = 100 random features, so that ϕ(*x*)^⊤^ϕ(*y*)≈exp(−γ∥*x*−*y*∥^2^) in expectation. For WUSTL-EHMS-2020, we instead use a Nyström landmark-based map (Williams and Seeger, 2001) with *m* = 600 landmark components sampled from the training data,


$$\phi \left(x\right)=W^{-1/2}k\left(x,z_{1}\right),\ldots ,k$$ (4)

where *W*_*ij*_ = *k*(*z*_*i*_, *z*_*j*_), ${\left\{z_{i}\right\}}_{i=1}^{m}$ are the landmark points, and *k* is the RBF kernel. These two feature-map families (γ = 1/RBFSampler, *m* = 100, for ECU-IoHT vs. γ = 0.1/Nyström, *m* = 600, for WUSTL-EHMS-2020) are used specifically for the *RFK a priori and matched-search* results reported in Sections 4.1 and 4.2, reflecting each original per-dataset script's own design rather than a single fixed architecture. The landmark-selection comparison itself (Section 3.6) is a separate experiment on both datasets: because landmark choice is specifically a Nyström concept, that comparison builds its own Nyström map for *both* ECU-IoHT and WUSTL-EHMS-2020, reusing each dataset's own γ and *m* from above as shared kernel hyperparameters, rather than reusing ECU-IoHT's RBFSampler map from this subsection. Sections 4.1–4.2's RFK results and Section 4.3's landmark-selection results are therefore two distinct experiments sharing the same per-dataset γ/*m* hyperparameters but not the same feature-map family on ECU-IoHT so that the landmark-selection comparison could be run identically on both datasets.

By Cover's theorem on pattern separability, projecting data into a sufficiently high-dimensional space makes it more likely that classes that overlap in the original space become linearly separable (Cover, 1965). This geometric intuition motivates the random-feature/Nyström construction, but we treat it as a plausibility argument for *why* a linear classifier can work well after this mapping, not as a guarantee for this specific data. We let Section 4's empirical results speak to the actual outcome rather than asserting linear separability without evidence.

### 3.6 Landmark selection: uniform, *K*-means, approximate ridge-leverage-score, and QI-AWNS

Equation 4's Nyström map requires choosing *m* landmark points ${\left\{z_{i}\right\}}_{i=1}^{m}$ from (or derived from) the training partition. We compare four landmark-selection strategies under otherwise-identical kernel hyperparameters (γ, *m*) and a shared LogisticRegression classifier (regularization *C* fixed per dataset to each dataset's *a priori* value, Section 3.7), so that the landmark-selection strategy is the only varying factor. All four strategies select from the same stratified random candidate pool of size min(5000, *n*_train_) drawn from the training partition (never validation or test); this pool restriction is a disclosed scalability simplification (Section 6), not a methodological difference among the strategies being compared.

**Uniform**. The scikit-learn default: *m* candidate-pool points are drawn uniformly at random without replacement.

*K***-means**. Following Zhang et al. (2008), we run *K*-means (specifically its mini-batch variant for tractability at the candidate-pool sizes used here) with *m* clusters on the candidate pool and use the *m* cluster centroids as landmarks. Centroids are synthetic points, not necessarily training-set rows, and the Nyström kernel evaluation in Equation 4 accommodates them without modification.

**Approximate ridge-leverage-score sampling**. Following Alaoui and Mahoney (2015), we draw a candidate subset *S* of size min(|pool|, max(3*m, m*+10)), compute its kernel matrix *K*_*SS*_, and approximate each point's ridge leverage score as


$$\begin{array}{c} \ell_{i}={\left(K_{SS}{\left(K_{SS}+\lambda I\right)}^{-1}\right)}_{ii}, \end{array}$$ (5)

with λ set to one-tenth of *K*_*SS*_'s average diagonal value (a single-pass, non-recursive approximation of the leverage scores, disclosed as a simplification relative to the full recursive algorithm in Alaoui and Mahoney (2015)); *m* landmarks are then sampled from *S* without replacement, with probability proportional to ℓ_*i*_.

**QI-AWNS (proposed)**. For each candidate-pool point *x*_*i*_ with label *y*_*i*_, we compute four importance components from the training partition only:

- *Classifier uncertainty* *u*_*i*_: the normalized binary entropy −*p*_*i*_log*p*_*i*_−(1−*p*_*i*_)log(1−*p*_*i*_) of *p*_*i*_ = Pr(*y* = 1∣*x*_*i*_) under a LogisticRegression fit on the full training partition.
- *Boundary distance* *b*_*i*_: 1/(1+|margin_*i*_|), where margin_*i*_ is the same classifier's signed decision-function value at *x*_*i*_ (larger *b*_*i*_ = closer to the decision boundary).
- *Representativeness* *r*_*i*_: $1/\left(1+{\bar{d}}_{i}\right)$, where ${\bar{d}}_{i}$ is the mean distance from *x*_*i*_ to its 10 nearest neighbors within the training partition (larger *r*_*i*_ = denser local neighborhood).
- *Minority-class importance* *m*_*i*_: 1−(count(*y*_*i*_)/*n*_train_), the fraction of *other*-class training samples, so the rarer class receives a larger value.

Each component is min-max normalized to [0, 1] across the candidate pool, then combined into a single importance score,


$$\begin{array}{c} s_{i}=\lambda_{u}u_{i}+\lambda_{b}b_{i}+\lambda_{r}r_{i}+\lambda_{m}m_{i}, \end{array}$$ (6)

with λ_*u*_+λ_*b*_+λ_*r*_+λ_*m*_ = 1. We then encode *s*_*i*_ as a normalized quantum-style amplitude and sample landmarks according to the squared amplitude, i.e. under a Born-rule-style measurement interpretation:


$$\begin{array}{c} a_{i}=\frac{\sqrt{s_{i}+\epsilon }}{\sqrt{\underset{j}{\sum }\left(s_{j}+\epsilon \right)}},\;p_{i}=a_{i}^{2}, \end{array}$$ (7)

with ϵ = 10^−8^ preventing a zero-probability point from being permanently excluded. *m* landmarks are then sampled from the candidate pool without replacement according to {*p*_*i*_}.

*We disclose the following algebraic identity explicitly, rather than let it remain implicit*: substituting Equation 6 into Equation 7 gives


$$\begin{array}{c} p_{i}=a_{i}^{2}=\frac{s_{i}+\epsilon }{\underset{j}{\sum }\left(s_{j}+\epsilon \right)}, \end{array}$$ (8)

i.e., squaring an *L*_2_-normalized amplitude vector recovers exactly the linear (score-proportional) normalization of *s*_*i*_ into a probability simplex, regardless of the intermediate square-root step. The amplitude language is therefore a genuine *interpretive* contribution: sampling as if measuring a normalized quantum state whose basis amplitudes encode importance, rather than a numerically distinct algorithm from directly normalizing *s*_*i*_ by its sum. We consider the four-component importance scoring (Equation 6) and this Born-rule-style sampling interpretation to be QI-AWNS's actual contributions; Section 4.3's ablation includes a variant (*full-deterministic*) that replaces probabilistic sampling under *p*_*i*_ with deterministic selection of the *m* highest-scoring points. This ablation isolates whether stochastic Born-rule-style sampling, rather than the combined importance score itself, contributes anything measurable.

**What “quantum-inspired” does, and does not, mean here**. We use this term, and the “QI” in QI-AWNS, strictly to describe a classical algorithm whose sampling step is *formulated* using the vocabulary of quantum-state amplitudes and Born-rule measurement, following an established line of “quantum-inspired” classical algorithms that borrow this vocabulary without requiring quantum hardware or claiming a computational speedup (Huynh et al., 2023; Tang, 2019) (Section 2.1). Equation 8 explicitly rules out any advantage attributable to the amplitude construction itself: there is no quantum hardware, no superposition, no entanglement, and every experiment in this study (Section 4) ran entirely on classical CPU hardware. We retain the amplitude formulation as an optional interpretive layer, not because it changes any sampling probability—Equation 8 shows it does not, since the square-root amplitude construction followed by squaring recovers exactly the direct score-proportional distribution–but because it names the specific reparameterization used and links it to the broader dequantization literature (Tang, 2019), which asks precisely when a proposed quantum algorithm's advantage can be matched classically. We therefore describe QI-AWNS throughout this paper as a task-aware importance-sampling mechanism with an optional, disclosed quantum-amplitude reparameterization—not a core methodological feature or source of novelty–and resist any phrasing implying quantum computational advantage, since Equation 8 shows none exists.

The combination weights (λ_*u*_, λ_*b*_, λ_*r*_, λ_*m*_) are chosen, per dataset, via a small grid search (10 candidate weight vectors, including each single component in isolation and several convex combinations) and scored only on validation balanced accuracy (never on test); Section 4.3 reports both the selected weights and the validation-tuning overfitting effect this search exposes on WUSTL-EHMS-2020.

#### 3.6.1 Time and memory complexity

We complement Section 4.5's empirical timing with the asymptotic complexity each landmark-selection strategy adds on top of the shared Nyström machinery, since wall-clock numbers alone (Table 3) do not show how each method would scale beyond the candidate-pool size |pool| = min(5000, *n*_train_) used here. Let *n* denote the training-partition size, *d* the raw feature dimensionality, *m* the number of landmarks, and |pool| the candidate-pool size defined in Section 3.6.

**Uniform sampling**. Selection takes *O*(|pool|) time (a single reservoir/random-choice pass) and *O*(*md*) memory to store the landmarks, independent of any scoring computation.

*K***-means**. Mini-batch *K*-means selection costs *O*(*tbmd*) time, where *t* is the number of mini-batch iterations and *b* is the mini-batch size, because each iteration assigns *b* points to their nearest of *m* centroids in *d* dimensions. Memory is *O*(*md*) for the centroids plus *O*(*bd*) for the current mini-batch, both independent of |pool|.

**Approximate ridge-leverage-score sampling**. Computing *K*_*SS*_ for the size-min(|pool|, max(3*m, m*+10)) candidate subset *S* requires *O*(|*S*|^2^*d*) time for the kernel matrix and *O*(|*S*|^3^) time for the matrix inversion in Equation 5, with *O*(|*S*|^2^) memory to store *K*_*SS*_; this is the most expensive of the four strategies, and the candidate-set size |*S*| is deliberately kept small (a small constant multiple of *m*) rather than equal to |pool|.

**QI-AWNS (proposed)**. The four importance components (Section 3.6) are each *O*(|pool|*d*) to *O*(|pool|*d*+|pool|*kd*): fitting the shared LogisticRegression classifier used for the uncertainty and boundary-distance components is *O*(|pool|*d*) per iteration under lbfgs; the representativeness component's *k*-nearest-neighbor query (*k* = 10) is *O*(|pool|*d*) per query under a brute-force search, so *O*(|pool|^2^*d*) for all candidate points, the dominant term for large pools (a *k*-d tree or ball tree would reduce this to *O*(|pool|log|pool|*d*), an optimization we did not implement here and note as a scalability lever); and the minority-class-importance component is *O*(|pool|), a single count. The amplitude encoding (Equation 7) and the final weighted-sampling draw are both *O*(|pool|). Memory is *O*(|pool|*d*) to hold the candidate pool's feature vectors plus the four *O*(|pool|) component-score arrays, and *O*(*md*) for the sampled landmarks themselves. QI-AWNS is therefore the only one of the four strategies with an *O*(|pool|^2^*d*) term (from the nearest-neighbor component), which is consistent with, and offers a structural explanation for, its measured fit time being the highest of the four at both datasets' current pool sizes (Table 3, 0.99 s vs. 0.54–0.80 s on ECU-IoHT); at the |pool| ≤ 5000 scale used throughout this paper this term remains small in absolute terms, but it is the term that would dominate cost growth if the candidate-pool restriction (Section 6) were relaxed toward the full training-partition size.

**Downstream Nyström transform and classifier (shared across all four strategies)**. Once landmarks are chosen, computing the Nyström feature map ϕ(*x*) for the *n* training points takes *O*(*nmd*) time (kernel evaluation against *m* landmarks) plus a one-time *O*(*m*^3^) eigendecomposition of the *m*×*m* landmark kernel matrix (Equation 4); fitting the linear classifier on the resulting ℝ^*m*^ embeddings is *O*(*nm*) per solver iteration, and inference on one sample is *O*(*md*) for the feature map plus *O*(*m*) for the linear decision function. Because *m*∈{100, …, 600} is fixed and small relative to *n* in both datasets (Section 3.1), this downstream cost, not the landmark-selection strategy itself, dominates total training time, which is why Table 3 shows all four strategies within a small constant factor of one another on fit time, latency, and memory despite their different asymptotic selection costs above: the selection step is a small additive term against an *O*(*nmd*) backdrop shared by all four.

### 3.7 Linear classification

The linear classifier trained on ϕ(*x*) is not uniformly a Linear Support Vector Classifier across datasets: direct inspection of the per-dataset training scripts shows that ECU-IoHT uses LinearSVC (scikit-learn defaults, *C* = 1), while WUSTL-EHMS-2020 uses LogisticRegression (*C* = 100, solver=‘lbfgs'); this is a genuine difference in classifier family between datasets, not a typo, and we document and reproduce it faithfully. Neither classifier uses class_weight=‘balanced' in this *a priori* configuration, unlike the tabular baselines described in Section 3.9; we preserve this as-is, since changing it would stop reproducing the pipeline's original behavior. (Section 3.6's landmark-selection comparison uses a shared LogisticRegression classifier for all four landmark strategies compared there, a deliberate, disclosed departure from this per-dataset *a priori* classifier choice, made specifically to isolate landmark-selection strategy as the only varying factor in that comparison.) For the linear SVC, given expanded feature vectors ϕ(*x*)∈ℝ^*m*^ and labels *y*_*i*_∈{−1, +1}, training solves the following optimization problem (Equation 9):


$$\begin{array}{c} \underset{w,b,\xi }{\min}\;\frac{1}{2}∥w{∥}^{2}+C\underset{i}{\sum }\xi_{i}\;\text{s.t. }y_{i}\left(w^{⊤}\phi \left(x_{i}\right)+b\right)\geq 1-\xi_{i},\;\xi_{i}\geq 0, \end{array}$$ (9)

where *w* is the separating hyperplane's normal vector, *b* is the bias, ξ_*i*_ are slack variables permitting soft-margin misclassification, and *C* trades margin width against training error (Cortes and Vapnik, 1995). LinearSVC does not produce calibrated probabilities; on ECU-IoHT, predictions are thresholded at decision_function = 0, and we use decision_function scores for ROC-AUC. LogisticRegression, used on WUSTL-EHMS-2020, produces calibrated probabilities via the logistic link (Cox et al., 1958); there we threshold at *p* = 0.5 and use predict_proba scores for ROC-AUC, consistent with each classifier's native interface.

The reasons this construction can be attractive for edge deployment are worth stating plainly: a linear classifier's generalization behavior is governed by margin rather than raw input dimensionality, so it does not automatically incur a curse-of-dimensionality penalty from the expanded ϕ(*x*)∈ℝ^*m*^ representation; fitting a linear decision boundary to precomputed embeddings converges quickly compared with iterative deep-network training; and the feature map itself, once its random directions or landmark points are fixed, is a cheap, parallelizable transformation suitable for low-power inference hardware. We treat these as reasons to rigorously evaluate the construction, not as a substitute for the empirical evaluation in Section 4.

### 3.8 Matched-budget hyperparameter search and fully nested cross-validation

The feature-map method, γ, embedding dimension *m*, classifier regularization *C*, and feature-selection percentile were fixed *a priori* in the original per-dataset scripts, before any experiment was run; since they were not selected via any validation-set search, they cannot reflect test-set leakage, but we also cannot rule out that a matched search would change the reported accuracy in either direction. To close this gap, we ran a validation-only random search over the model's own hyperparameters, namely the feature-map method, γ, embedding dimension, classifier regularization *C*, feature-selection percentile, and the classifier family itself (LinearSVC vs. LogisticRegression), using a fixed 30-evaluation budget for both datasets. Each candidate is fit on the training partition and scored on the validation partition only, exactly once; the best candidate by validation accuracy is refit on the training partition and evaluated exactly once on the held-out test partition, following the same discipline as item 5 of Section 3.2. We also ran the fully nested 5-fold cross-validation described in Section 3.2, item 6, using each dataset's correct *a priori* classifier (LinearSVC for ECU-IoHT, LogisticRegression for WUSTL-EHMS-2020). Results for both are reported in Section 4.

### 3.9 Tabular baseline models

To test directly whether the Nyström/random-feature construction earns its added conceptual complexity relative to off-the-shelf classifiers, we add five tabular baselines evaluated under the identical leakage-free protocol (same split, same training-only feature selection and scaling): logistic regression (Cox et al., 1958), a random forest (Breiman, 2001) (200 trees), XGBoost (Chen and Guestrin, 2016) (300 boosting rounds, max depth 6), a plain full-kernel RBF support-vector classifier (Cortes and Vapnik, 1995), and a multilayer perceptron (Rumelhart et al., 1986) (two hidden layers, 64 and 32 units), all implemented with scikit-learn (Pedregosa et al., 2011) except XGBoost's dedicated library. All baselines except the MLP use class_weight=~balanced~ (or XGBoost's scale_pos_weight) computed from training labels only; MLPClassifier does not support per-sample or per-class weighting in scikit-learn, which we report as a known asymmetry. Baseline hyperparameters were fixed by convention rather than searched, matching the RFK model's *a priori* search-effort level rather than its matched-search level; we note this as a further, minor search-budget asymmetry (Section 6).

### 3.10 Statistical evaluation protocol

For each dataset and model, we report accuracy, balanced accuracy, precision, recall, F1-score, specificity, false-positive rate, Matthews correlation coefficient, ROC-AUC, PR-AUC, a Wilson-score 95% confidence interval on accuracy (Wilson, 1927) (more reliable than the normal-approximation interval for proportions near 0 or 1), fit time, and per-sample inference latency. Specifically, for the landmark-selection comparison (Section 3.6, Section 4.3) specifically, we additionally report: (i) McNemar's exact test (McNemar, 1947) on paired test-set predictions at a single split (seed 42), testing whether QI-AWNS and a given baseline landmark strategy disagree asymmetrically on which test samples they classify correctly; and (ii) a Wilcoxon signed-rank test over 10 reseeded train/validation/test splits (seeds 42, 1–9), run under the two weight-tuning protocols defined in Section 4.3, testing whether QI-AWNS's balanced accuracy is systematically higher or lower than a given baseline's across reseeds under each protocol; this is a stronger claim than a single-split comparison, since it is not vulnerable to one split's particular sampling variance. All computations were performed with scikit-learn, scipy, and statsmodels.

## 4 Experimental results

The primary experimental contribution of this study is the landmark-selection comparison in Section 4.3, which isolates QI-AWNS against uniform, *K*-means, and approximate ridge-leverage-score sampling under matched hyperparameters, 10 reseeded splits, and two evaluation protocols. Section 4.3's ablation and weight-search results are likewise part of this primary contribution. Sections 4.1 and 4.2 report the underlying Nyström/random-feature (RFK) model's *a priori* and matched-search configurations and the tabular-baseline comparison. We retain these as supporting context that establishes the pipeline's leakage-free numbers and the accuracy gap against unsearched classical baselines, but they are not this paper's central claim. A reader most interested in the QI-AWNS result may proceed directly to Section 4.3.

### 4.1 ECU-IoHT

Table 4 reports test-set performance on ECU-IoHT (*n* = 16, 682). With hyperparameters fixed *a priori* (RBFSampler, γ = 1, *m* = 100, LinearSVC, no class weighting), the model achieves 93.27% accuracy (95% CI [92.88, 93.64]), precision 99.98%, recall 91.49%, F1 95.55%, AUC 0.9922, and fits in 15.69 s (Figure 2). The matched, 30-evaluation validation-only search, now including the classifier family as a searched hyperparameter, raises test accuracy to 96.39% (precision 100.00%, recall 95.42%, F1 97.66%, AUC 0.9953), at a total cost of approximately 120.2 s (105.9 s search plus a 14.3 s final refit) (Figure 3). Fully nested 5-fold cross-validation yields a mean accuracy of 94.37% (std 1.85% across folds; fold scores 98.08%, 93.44%, 93.39%, 93.54%, 93.41%), consistent with and slightly above the *a priori* single-split test accuracy.

**Table 4:** ECU-IoHT test-set results (*n* = 16, 682; attack-class Prec./Rec./F1).

Model	Acc. (%)	95% CI	Prec.	Rec.	F1	AUC
RFK (a priori)	93.27	[92.88, 93.64]	99.98	91.49	95.55	0.9922
RFK (matched search)	**96.39**	[96.09, 96.66]	100.00	95.42	97.66	0.9953
Logistic Regression	91.94	[91.52, 92.35]	98.36	91.31	94.71	0.9350
Random Forest	98.29	[98.08, 98.47]	98.57	99.26	98.92	0.9969
XGBoost	98.27	[98.06, 98.45]	98.57	99.24	98.91	0.9969
RBF-SVM (full kernel)	93.29	[92.90, 93.66]	100.00	91.50	95.56	0.9934
MLP (64,32)	98.19	[97.98, 98.38]	98.57	99.14	98.86	0.9965

Bold indicates the higher-accuracy configuration between this study's two RFK (Random-Feature Kernel) settings, the a priori and matched-search hyperparameter configurations.

**Figure 2:**
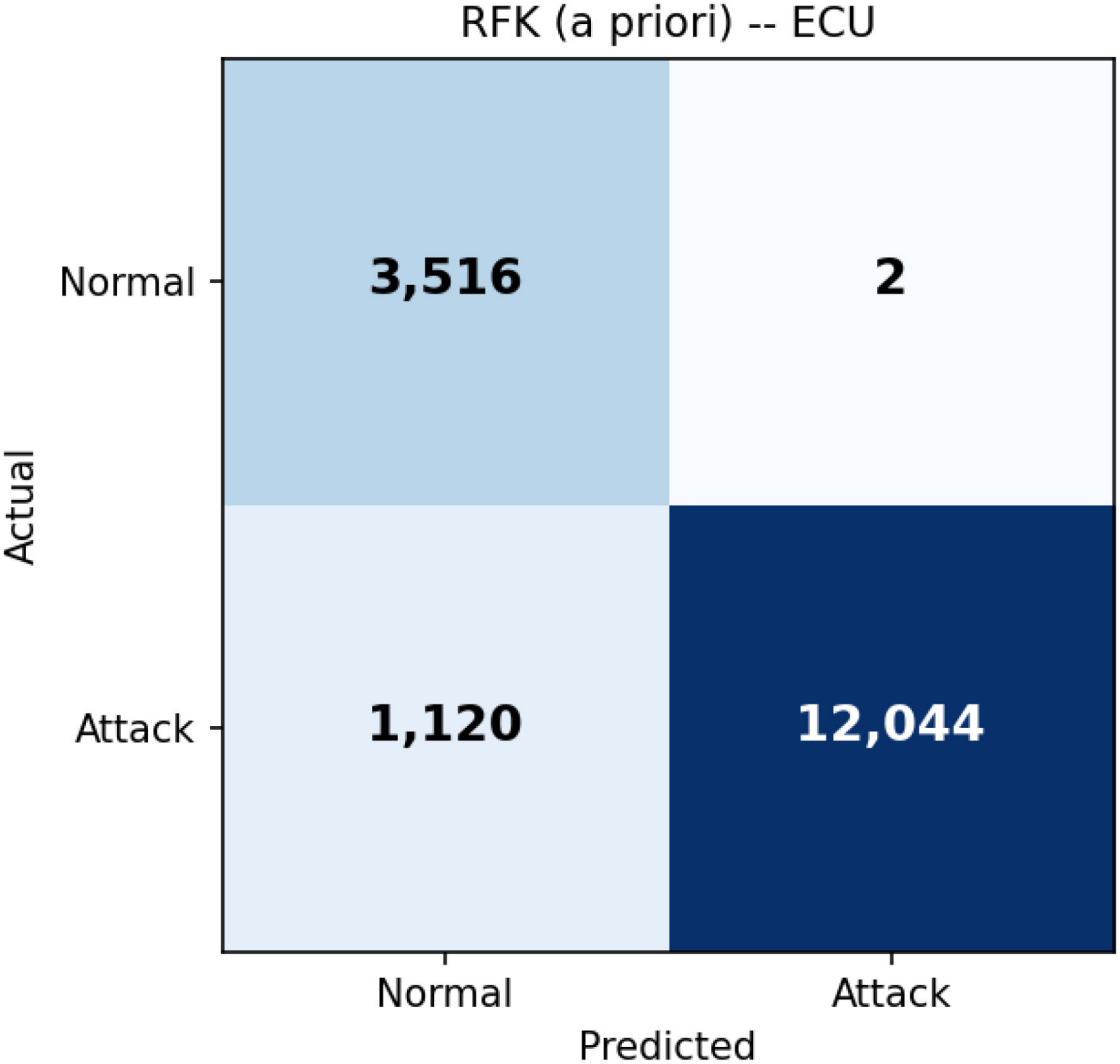
Confusion matrix, ECU-IoHT test set, RFK (a priori configuration). The matched-search configuration's confusion matrix is visually near-identical at this scale and is provided as Supplementary material S1 rather than repeated here.

**Figure 3:**
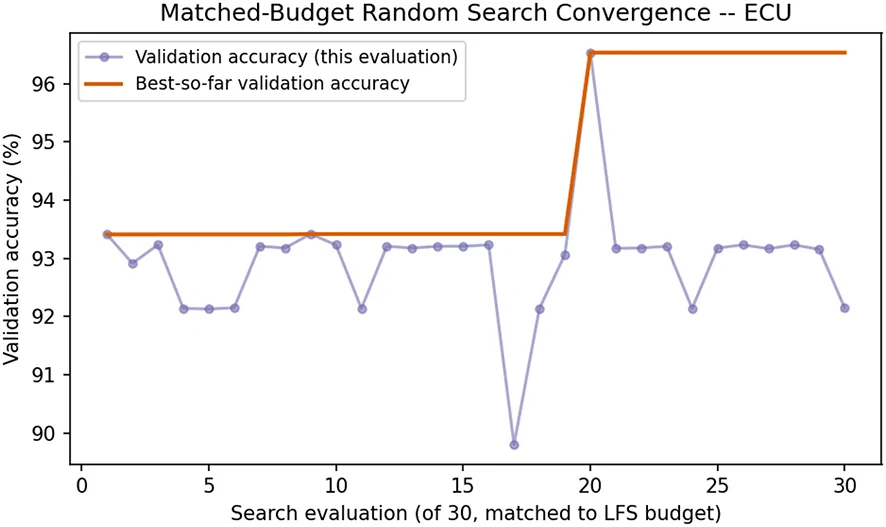
Matched-budget random-search convergence, ECU-IoHT (30 evaluations, real per-evaluation validation accuracy and running best-so-far).

Notably, random forest, XGBoost, and the MLP baseline all exceed the RFK model's matched-search accuracy on ECU-IoHT by roughly 1.8–1.9 percentage points, using conventional, off-the-shelf implementations with an unsearched, *a priori* configuration. We report this plainly: on this dataset, the Nyström/random-feature construction's chief empirical advantage is training speed and model compactness (Section 5), not a raw-accuracy edge over simple tabular baselines. (We use “RFK,” short for Random-Feature Kernel, for this legacy RBFSampler/LinearSVC-family model throughout Section 4.1 and Section 4.2 specifically to avoid confusion with QI-AWNS, the paper's actual proposed method, introduced separately in Section 4.3).

### 4.2 WUSTL-EHMS-2020

Table 5 reports test-set performance on WUSTL-EHMS-2020 (*n* = 2, 448; 2,141 normal, 307 attack). With hyperparameters fixed *a priori* (Nyström, γ = 0.1, *m* = 600, LogisticRegression, *C* = 100, no class weighting), the model reaches 92.89% accuracy (95% CI [91.81, 93.84]), precision 92.90%, recall 46.91%, F1 62.34%, AUC 0.8817, fit in 1.07 s (Figure 4). The matched, 30-evaluation search, again including classifier family as a searched hyperparameter, *lowers* test accuracy here to 87.95% (precision 51.92%, recall 52.77%, F1 52.34%, AUC 0.8342), at a total cost of approximately 38.5 s (Figure 5). Fully nested 5-fold cross-validation gives mean accuracy 92.99% (std 0.44%; fold scores 93.37%, 93.11%, 92.79%, 93.44%, 92.25%), closely matching the *a priori* single-split test accuracy. Table 6 collects both datasets' per-fold scores in one place for easier verification against the raw result files (results/nested_cv_ecu.json, results/nested_cv_wustl.json) than the running-text form above.

**Table 5:** WUSTL-EHMS-2020 test-set results (*n* = 2, 448; attack-class Prec./Rec./F1).

Model	Acc. (%)	95% CI	Prec.	Rec.	F1	AUC
RFK (a priori)	**92.89**	[91.81, 93.84]	92.90	46.91	62.34	0.8817
RFK (matched search)	87.95	[86.60, 89.18]	51.92	52.77	52.34	0.8342
Logistic Regression	87.66	[86.30, 88.91]	50.85	48.53	49.67	0.8088
Random Forest	92.97	[91.89, 93.92]	92.45	47.88	63.09	0.8876
XGBoost	89.71	[88.44, 90.85]	56.63	76.55	65.10	0.9402
RBF-SVM (full kernel)	89.38	[88.10, 90.54]	58.13	54.72	56.38	0.8474
MLP (64,32)	93.50	[92.46, 94.41]	88.54	55.37	68.14	0.8990

Bold indicates the higheraccuracy configuration between this study's two RFK settings, the a priori and matched-search hyperparameter configurations. In this dataset, the a priori configuration achieves the higher value.

**Figure 4:**
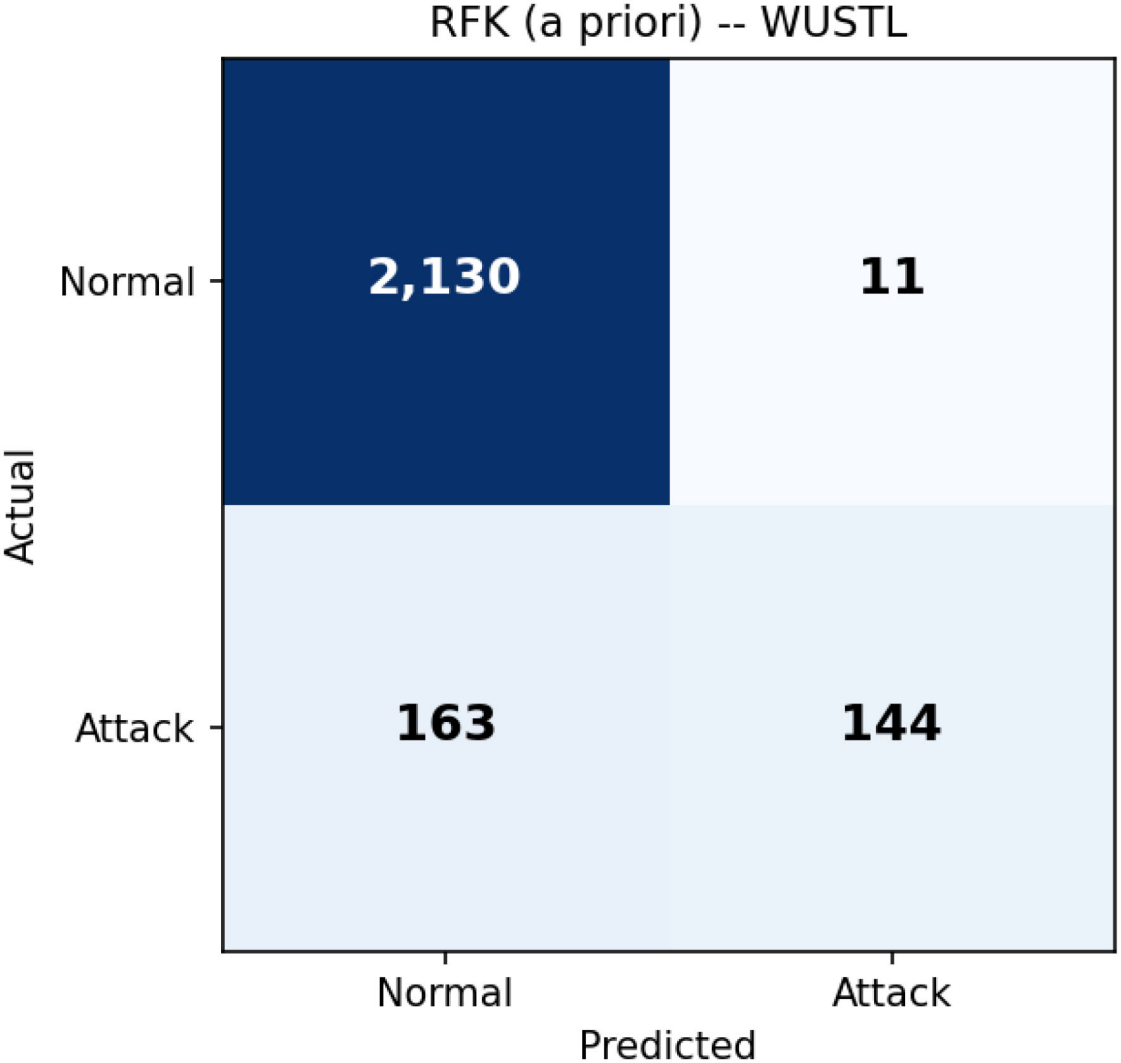
Confusion matrix, WUSTL-EHMS-2020 test set, RFK (a priori configuration). The matched-search configuration's confusion matrix is provided as Supplementary material S1 rather than repeated here.

**Figure 5:**
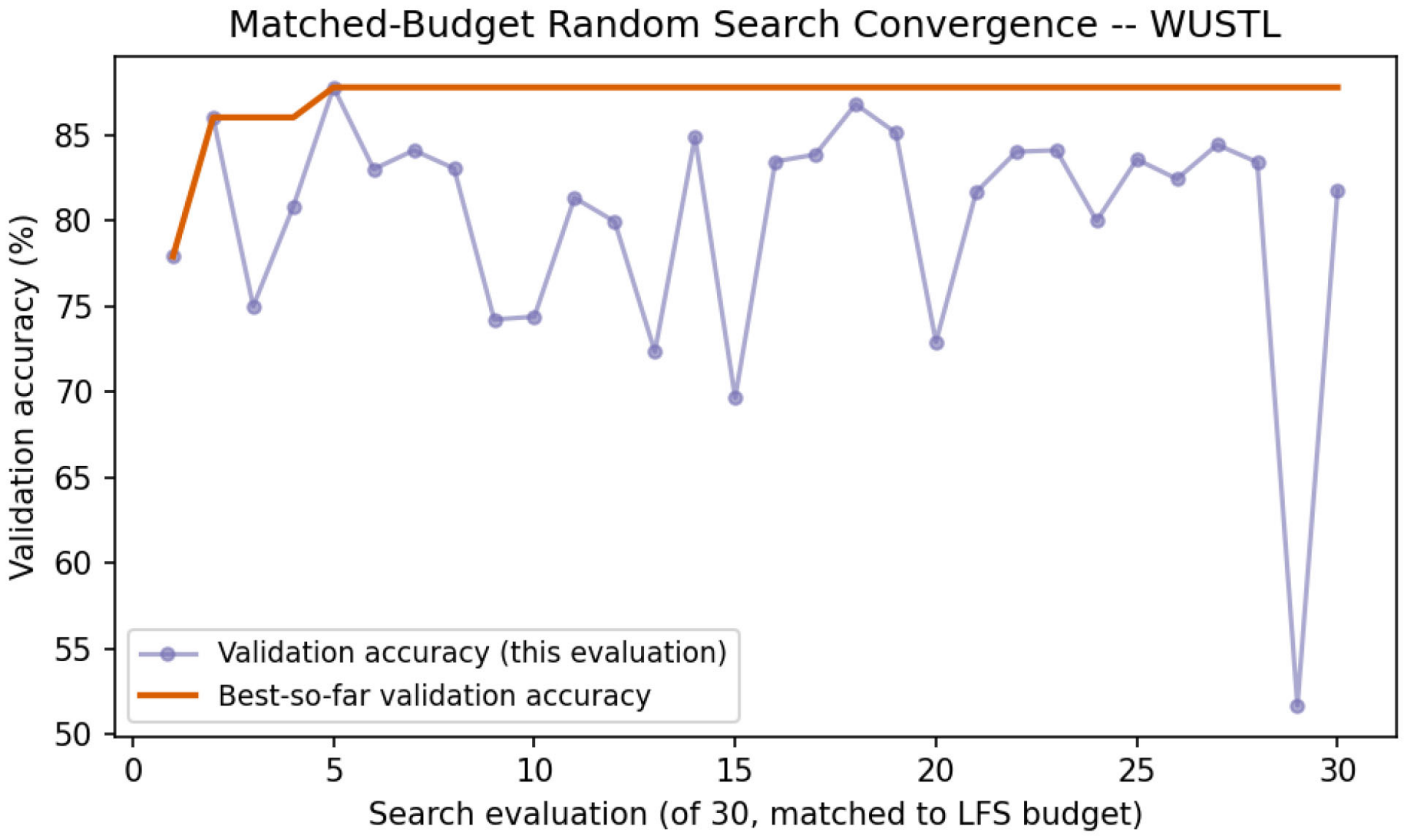
Matched-budget random-search convergence, WUSTL-EHMS-2020 (30 evaluations).

**Table 6:** Fully nested 5-fold cross-validation: per-fold test accuracy (%).

Dataset	Fold 1	Fold 2	Fold 3	Fold 4	Fold 5	Mean	Std
ECU-IoHT	98.08	93.44	93.39	93.54	93.41	94.37	1.85
WUSTL-EHMS-2020	93.37	93.11	92.79	93.44	92.25	92.99	0.44

Random forest (92.97%) and the MLP baseline (93.50%) are essentially tied with, or slightly exceed, the RFK model's best (*a priori*) result here, again using unsearched, conventional configurations. The RFK model's low attack-class recall (46.91% at the *a priori* configuration) is the more clinically relevant weakness: at this operating point the model misses more than half of true attacks in our test partition, a precision-favoring trade-off we revisit in Section 5.

**Class weighting vs. SMOTE oversampling**. All results above use class_weight=~balanced~ to address WUSTL-EHMS-2020's 12.5% attack-class imbalance (Section 3.1); we also test whether SMOTE oversampling (Chawla et al., 2002) performs better. We compare the two directly on the matched-search, balanced-accuracy-scored configuration (Nyström, γ = 0.2863, *m* = 600, LogisticRegression, *C* = 0.6701, percentile 60; Section 3.8), holding feature selection, scaling, and the kernel approximation identical between both variants so only the imbalance-handling step differs: (A) class weighting on the real (non-resampled) transformed training features, reproducing this paper's existing approach exactly (84.68% accuracy, 74.27% recall, matching Table 5's balanced-accuracy-scored search result); and (B) SMOTE applied to the transformed training features only, after the kernel approximation is fit on the true (non-synthetic) training distribution, so synthetic points never influence landmark placement, followed by a classifier under scikit-learn's default (uniform) weighting. Table 7 reports both. SMOTE is marginally better than class weighting across every metric on this single split (e.g., recall +0.32 points, F1 +0.38 points, MCC +0.0045), but the margin is small relative to the differences already documented between search protocols and baselines elsewhere in this paper, and we evaluated this comparison on one split rather than the 10-reseed protocol used for the landmark-selection comparison (Section 3.10). Therefore, we do not treat this as a statistically established advantage for SMOTE, only as evidence that resampling is, at minimum, a competitive alternative to class weighting here and does not clearly underperform it.

**Table 7:** WUSTL-EHMS-2020: class weighting vs. SMOTE (matched-search config., single split).

Imbalance handling	Acc. (%)	Bal. Acc. (%)	Prec.	Rec.	F1	MCC
Class weighting (existing)	84.68	80.22	43.51	74.27	54.87	0.4880
SMOTE (uniform weighting)	**84.84**	**80.45**	**43.87**	**74.59**	**55.25**	**0.4925**

Bold indicates the imbalancehandling strategy, SMOTE oversampling, that achieved the higher value for each reported metric in this single-split comparison.

### 4.3 Landmark-selection comparison: QI-AWNS vs. uniform, *K*-means, and approximate ridge-leverage-score sampling

Table 8 reports the single-split (seed 42) test-set comparison of the four landmark-selection strategies (Section 3.6) under matched kernel hyperparameters and a shared LogisticRegression classifier. On ECU-IoHT, QI-AWNS (validation-tuned weights: representativeness-only, λ_*r*_ = 1) reaches the highest accuracy (93.18%) and balanced accuracy (95.68%) of the four, narrowly ahead of *K*-means and approximate ridge-leverage-score sampling (both 93.12%/95.64%) and uniform sampling (93.09%/95.62%). On WUSTL-EHMS-2020, QI-AWNS (validation-tuned weights: λ_*u*_ = 0.5, λ_*b*_ = 0.3, λ_*r*_ = 0.1, λ_*m*_ = 0.1) reaches 84.11% accuracy but the *lowest* balanced accuracy (78.64%) of the four, behind uniform (79.10%) and approximate ridge-leverage-score (79.06%) sampling.

**Table 8:** Landmark-selection comparison, single split (seed 42); attack-class Prec./Rec./F1.

Dataset	Method	Acc. (%)	Bal. Acc. (%)	Prec.	Rec.	F1	Spec.	AUC	PR-AUC
ECU-IoHT	Uniform Nyström	93.09	95.62	100.00	91.25	95.42	100.00	0.9902	0.9971
	*K*-means Nyström	93.12	95.64	100.00	91.29	95.44	100.00	0.9916	0.9974
	Approx. RLS Nyström	93.12	95.64	100.00	91.29	95.44	100.00	0.9915	0.9973
	QI-AWNS (proposed)	**93.18**	**95.68**	100.00	91.36	95.48	100.00	0.9911	0.9973
WUSTL-EHMS-2020	Uniform Nyström	84.19	**79.10**	42.37	72.31	53.43	85.89	0.8884	0.7101
	*K*-means Nyström	84.15	78.80	42.23	71.66	53.14	85.94	0.8902	0.7158
	Approx. RLS Nyström	83.62	79.06	41.33	72.96	52.77	85.15	0.8903	0.7144
	QI-AWNS (proposed)	**84.11**	78.64	42.12	71.34	52.96	85.94	0.8883	0.7092

“Approx. RLS” = approximate ridge-leverage-score sampling (Section 3.6), abbreviated here for column width; not the full recursive algorithm.

A single split, however, cannot determine whether these small differences reflect a genuine effect or sampling variance from that split. We report two distinct multi-seed protocols across 10 reseeded train/validation/test splits (seeds 42, 1–9) because they answer different questions. **Protocol A (frozen weights)** reuses the seed-42-tuned importance weights (Table 9) unchanged at every reseed; this evaluates a single, fixed, already-specified algorithm. **Protocol B (per-seed retuning)** reruns the full 10-candidate validation-only weight search independently within each seed's train/validation split, then evaluates that seed's retuned configuration on that seed's test partition; this evaluates the complete end-to-end training procedure, including weight selection, as a unit. Table 10 reports mean±std for both protocols.

**Table 9:** Validation-only importance-weight search: all 10 candidates.

(λ_*u*_, λ_*b*_, λ_*r*_, λ_*m*_)				Val. Bal. Acc.	
λ_*u*_	λ_*b*_	λ_*r*_	λ_*m*_	ECU	WUSTL
1.0	0.0	0.0	0.0	0.9429	0.7613
0.0	1.0	0.0	0.0	0.9416	0.7580
0.0	0.0	1.0	0.0	**0.9576**	0.7538
0.0	0.0	0.0	1.0	0.9534	0.7522
0.25	0.25	0.25	0.25	0.9535	0.7554
0.4	0.4	0.1	0.1	0.9429	0.7585
0.3	0.3	0.2	0.2	0.9523	0.7575
0.5	0.3	0.1	0.1	0.9429	**0.7626**
0.2	0.2	0.3	0.3	0.9534	0.7594
0.1	0.1	0.4	0.4	**0.9576**	0.7608

Bold indicates the highest validation balanced accuracy achieved among the ten candidate weight vectors evaluated, shown separately for each dataset column.

**Table 10:** 10-seed mean±Std (balanced accuracy / recall / F1, %).

Dataset	Method	Bal. Acc.	Rec.	F1
ECU-IoHT	Uniform	95.55 ± 0.45	91.19 ± 0.93	95.38 ± 0.51
	*K*-means	95.72 ± 0.18	91.48 ± 0.31	95.54 ± 0.17
	Approx. RLS	95.69 ± 0.15	91.47 ± 0.30	95.53 ± 0.16
	QI-AWNS, Protocol A (frozen)	94.76 ± 1.30	90.66 ± 1.06	94.95 ± 0.78
	QI-AWNS, Protocol B (retuned)	**95.74 ± 0.15**	**91.48 ± 0.31**	**95.55 ± 0.17**
WUSTL	Uniform	**78.11 ± 0.90**	70.68 ± 1.35	**52.10 ± 1.58**
	*K*-means	78.07 ± 1.04	70.65 ± 1.80	52.01 ± 1.65
	Approx. RLS	78.07 ± 1.18	70.46 ± 2.06	52.16 ± 1.83
	QI-AWNS, Protocol A (frozen)	78.25 ± 0.91	**71.17 ± 1.37**	52.08 ± 1.61
	QI-AWNS, Protocol B (retuned)	78.10 ± 1.12	70.68 ± 1.72	52.04 ± 1.78

Bold indicates the highest ten-seed mean value achieved among the five compared configurations, shown separately for each dataset block and column.

The two protocols diverge sharply on ECU-IoHT and not at all on WUSTL-EHMS-2020. Under Protocol A, QI-AWNS's 10-seed mean balanced accuracy (94.76%) is *lower* than all three baselines (95.55–95.72%), pulled down by two reseeds (seeds 4, 6, and 9) in which the frozen density-only weighting regressed clearly (e.g., 90.61% at seed 4 vs. 93.6% for the baselines). Under Protocol B, retuning weights per seed recovers each of these regressions (seed 4 rises to 93.70%), and QI-AWNS's mean (95.74%) becomes the highest of all five configurations on ECU-IoHT, narrowly ahead of *K*-means (95.72%) and approximate ridge-leverage-score (95.69%); a paired Wilcoxon test confirms that retuning helps significantly here (*p* = 0.0156). On WUSTL-EHMS-2020, Protocol B provides no such recovery (*p* = 0.25, not significant), and both protocols remain statistically indistinguishable from all three baselines.

Table 11 reports paired significance tests against each baseline (Section 3.10), with Holm-corrected *p*-values (in parentheses) across the predeclared six-test family of Wilcoxon comparisons run within each protocol; a more conservative 12-test family combining both protocols did not change any conclusion below (raw per-seed results in the code repository, Data Availability Statement). McNemar's test on the single seed-42 split shows QI-AWNS disagreeing favorably with uniform sampling on ECU-IoHT at a level unlikely under chance (14 vs. 0, *p* = 1.2 × 10^−4^), but this advantage weakens against the two non-uniform baselines (14 vs. 5, *p* = 0.064) and is not significant on WUSTL-EHMS-2020 against any baseline. The 10-seed Wilcoxon test is protocol-dependent: under Protocol A, QI-AWNS shows no significant advantage over any baseline on either dataset, and its apparent disadvantage against *K*-means on ECU-IoHT (raw *p* = 0.039) does not survive Holm correction (adjusted *p* = 0.234); under Protocol B, its raw advantage over uniform sampling on ECU-IoHT (*p* = 0.0215) likewise does not survive correction (adjusted *p* = 0.129). At α = 0.05, once the multiple-comparison family is accounted for, no comparison in Table 11 remains significant in either direction; we therefore treat the ECU-IoHT/Protocol-B/uniform result as a small, uncorrected pairwise finding, not a confirmed effect, throughout the paper. QI-AWNS remains indistinguishable from *K*-means and approximate ridge-leverage-score on ECU-IoHT under both protocols, and from all three baselines on WUSTL-EHMS-2020. Table 12 reports paired effect sizes, since a *p*-value alone does not convey magnitude: even the ECU-IoHT/Protocol-B/uniform comparison, the largest and most favorable in the study, is a mean paired difference of only 0.18 points (bootstrap 95% CI [0.03, 0.45]; 8 wins, 0 ties, 2 losses of 10; rank-biserial *r* = 0.78). We read this as a genuinely mixed, dataset- and protocol-dependent finding: (i) Protocol A's instability is real and reseed-dependent; (ii) Protocol B's retuning fixes it and produces a small, uncorrected-only advantage over the weakest baseline on ECU-IoHT specifically; (iii) QI-AWNS shows no advantage over the two non-uniform baselines on either dataset; and (iv) on WUSTL-EHMS-2020, no version of QI-AWNS beats any baseline.

**Table 11:** Paired significance tests, QI-AWNS vs. each baseline (10 seeds); Holm-Corrected *p* (6-test within-protocol family) in parentheses.

Dataset	Baseline	McNemar *p* (seed 42)	Wilcoxon *p*, Protocol A (Holm)	Wilcoxon *p*, Protocol B (Holm)
ECU-IoHT	Uniform	1.2 × 10^−4^	0.375 (0.930)	0.022 (0.129)
	*K*-means	0.064	0.039 (0.234)	0.547 (1.000)
	Approx. RLS	0.064	0.061 (0.303)	0.098 (0.488)
WUSTL	Uniform	0.868	0.385 (0.930)	0.826 (1.000)
	*K*-means	1.000	0.232 (0.930)	0.941 (1.000)
	Approx. RLS	0.188	0.275 (0.930)	0.820 (1.000)

**Table 12:** Paired effect sizes, QI-AWNS vs. each baseline (10 seeds, balanced accuracy, percentage points).

Dataset	Baseline (protocol)	Mean Δ	Median Δ	95% CI (paired bootstrap)	Wins/Ties/Losses (of 10)	Rank-Biserial *r*
ECU-IoHT	Uniform (A, frozen)	−0.80	N/A	[−1.78, 0.06]	4/0/6	−0.35
	*K*-means (A, frozen)	−0.96	N/A	[−1.91, −0.18]	1/2/7	−0.83
	Approx. RLS (A, frozen)	−0.93	N/A	[−1.79, −0.13]	2/0/8	−0.67
	Uniform (B, retuned)	+0.18	N/A	[0.03, 0.45]	8/0/2	+0.78
	*K*-means (B, retuned)	+0.02	N/A	[−0.01, 0.07]	4/2/4	+0.28
	Approx. RLS (B, retuned)	+0.05	N/A	[0.01, 0.10]	6/1/3	+0.64
WUSTL	Uniform (A, frozen)	+0.14	N/A	[−0.08, 0.38]	6/0/4	+0.31
	*K*-means (A, frozen)	+0.18	N/A	[−0.03, 0.43]	6/0/4	+0.46
	Approx. RLS (A, frozen)	+0.18	N/A	[−0.07, 0.43]	7/0/3	+0.42
	Uniform (B, retuned)	−0.02	N/A	[−0.28, 0.20]	5/0/5	+0.09
	*K*-means (B, retuned)	+0.02	N/A	[−0.20, 0.27]	5/0/5	−0.02
	Approx. RLS (B, retuned)	+0.03	N/A	[−0.20, 0.31]	3/1/6	−0.11

Protocol A: frozen weights; Protocol B: per-seed retuning (Section 4.3). *Direction matters here*: the ECU-IoHT/*K*-means Protocol A entry (raw *p* = 0.039) would be significant in QI-AWNS's *disfavor* at an uncorrected threshold (Protocol A's mean, 94.76%, is below *K*-means' 95.72%, Table 10), but it does not survive Holm correction; the ECU-IoHT/Uniform/Protocol-B entry (raw *p* = 0.022), the only comparison significant in QI-AWNS's favor at an uncorrected threshold, likewise does not survive Holm correction (adjusted *p* = 0.129). No entry in this table is significant after correction for multiple comparisons at α = 0.05.

Δ = QI-AWNS minus baseline, balanced accuracy, percentage points; positive favors QI-AWNS. 95% CI computed by paired bootstrap (10,000 resamples) over the 10 seed-level differences. Rank-biserial *r* is the matched-pairs effect size derived from the Wilcoxon signed-rank statistic (*r* = (*W*^+^−*W*^−^)/(*W*^+^+*W*^−^)), with |*r*| conventionally interpreted as small (~0.1–0.3), medium (~0.3–0.5), or large (>0.5); several entries here reach “large” *r* despite raw mean differences below one percentage point and Holm-corrected *p*-values above 0.05, illustrating why we report effect size and significance together rather than either alone. Median Δ is omitted (shown as N/A) because with only 10 paired observations the median is a single order statistic and adds no information beyond the mean and win/tie/loss counts already reported; the full per-seed differences are released in the public code repository as qiawns_effect_sizes.json (Data Availability Statement).

Figure 6 presents Table 12 as a forest plot because a table of point estimates and intervals is easy to scan row by row but hard to compare visually at a glance. Plotting each comparison's mean difference and 95% bootstrap CI against a reference line at zero immediately shows which comparisons' intervals exclude zero (a necessary, though not Holm-corrected-sufficient, condition for significance) and which do not.

**Figure 6:**
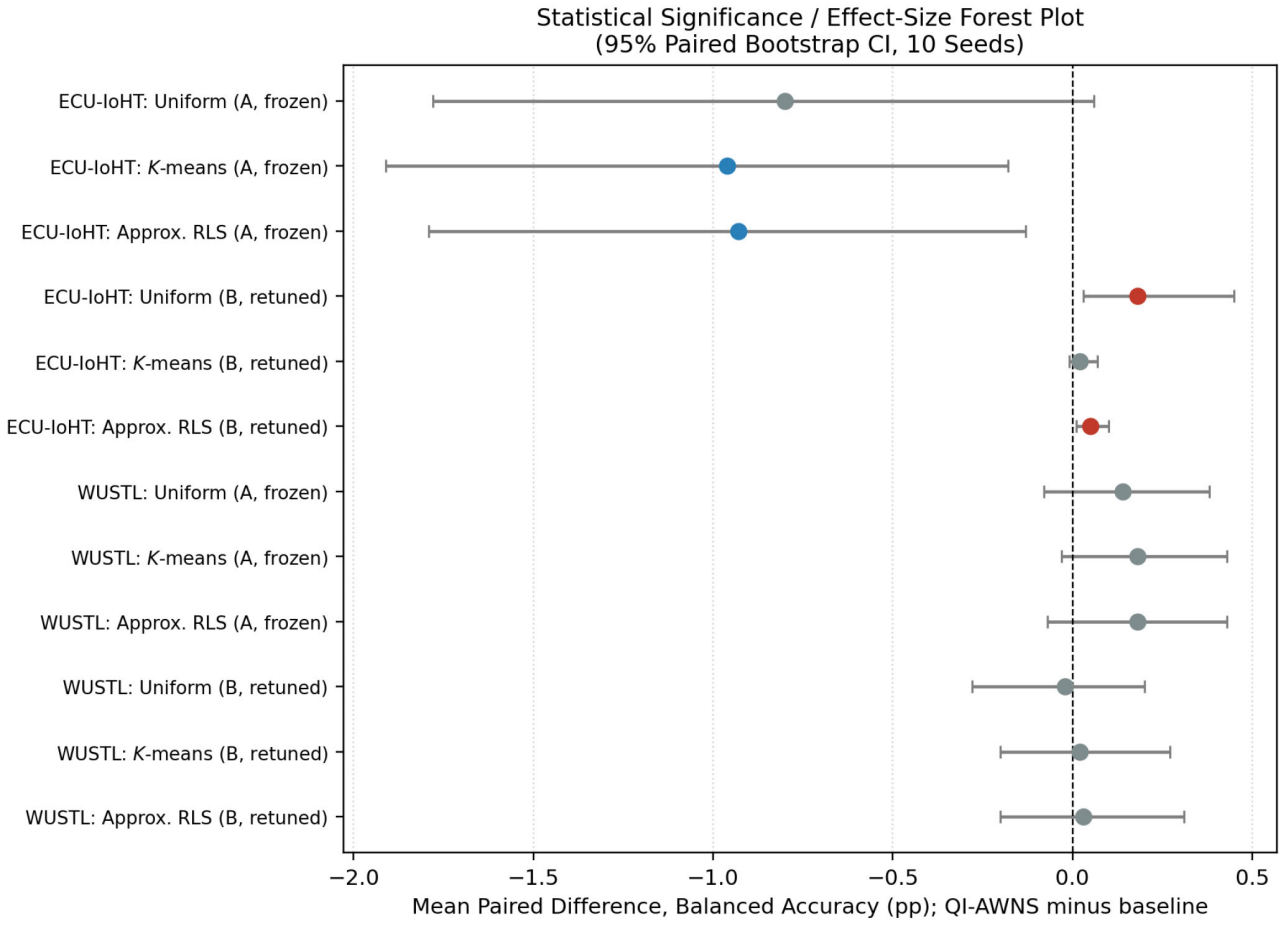
Forest plot of paired effect sizes (mean difference, balanced accuracy, with 95% paired-bootstrap CI), QI-AWNS vs. each baseline, both protocols, both datasets (Table 12). Points/intervals crossing zero indicate no directional effect; none of these comparisons remain significant after Holm correction (Table 11).

Figure 7 complements the balanced-accuracy/F1 view above with ROC-AUC and PR-AUC: the left panel places the four landmark-selection strategies alongside the five tabular baselines (Section 3.9) on ROC-AUC for both datasets, and the right panel isolates PR-AUC for the four landmark strategies (the metric most sensitive to class imbalance, relevant given WUSTL-EHMS-2020's 12.5% minority class, Section 3.1).

**Figure 7:**
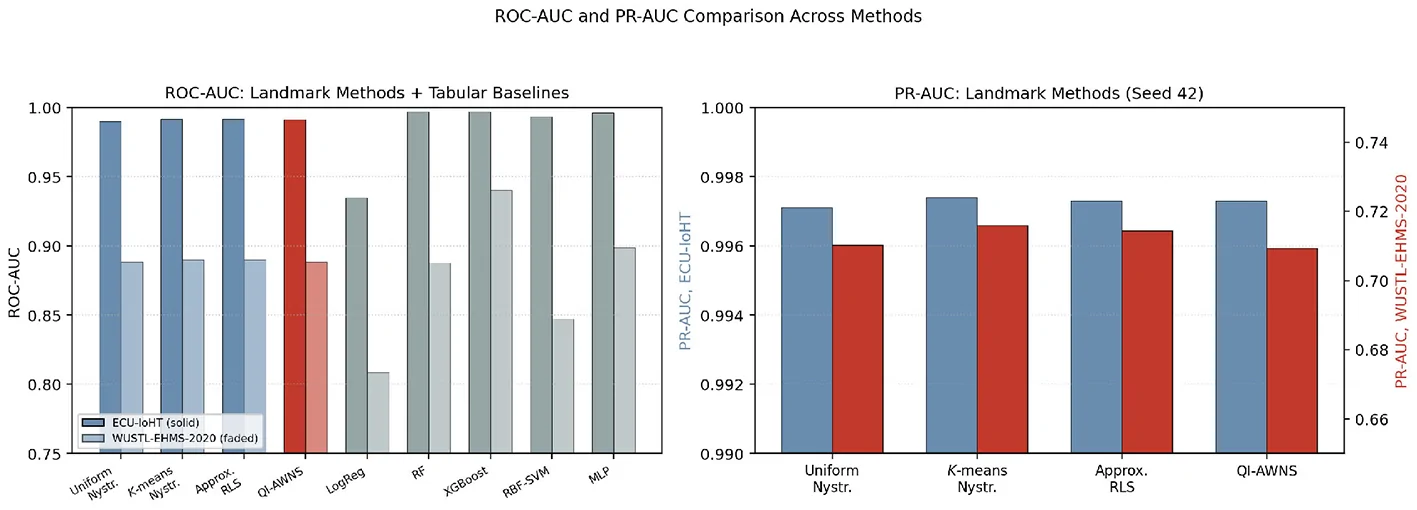
ROC-AUC (left, landmark methods and tabular baselines) and PR-AUC (right, landmark methods only, seed 42) across both datasets. Note the differing *y*-axis scales for WUSTL-EHMS-2020's PR-AUC (right panel, secondary axis), a lower-magnitude but still class-imbalance-sensitive metric on this dataset.

Figure 8 visualizes Table 10's 10-seed means directly, so the near-overlapping bars, rather than only the *p*-values above, convey how small the between-method differences are in absolute terms on both datasets: the four landmark-selection strategies cluster within roughly one percentage point of one another in both balanced accuracy and F1 on ECU-IoHT, and within a similarly narrow band on WUSTL-EHMS-2020, with error bars (one standard deviation across seeds) that visibly overlap across every pair of methods on both metrics.

**Figure 8:**
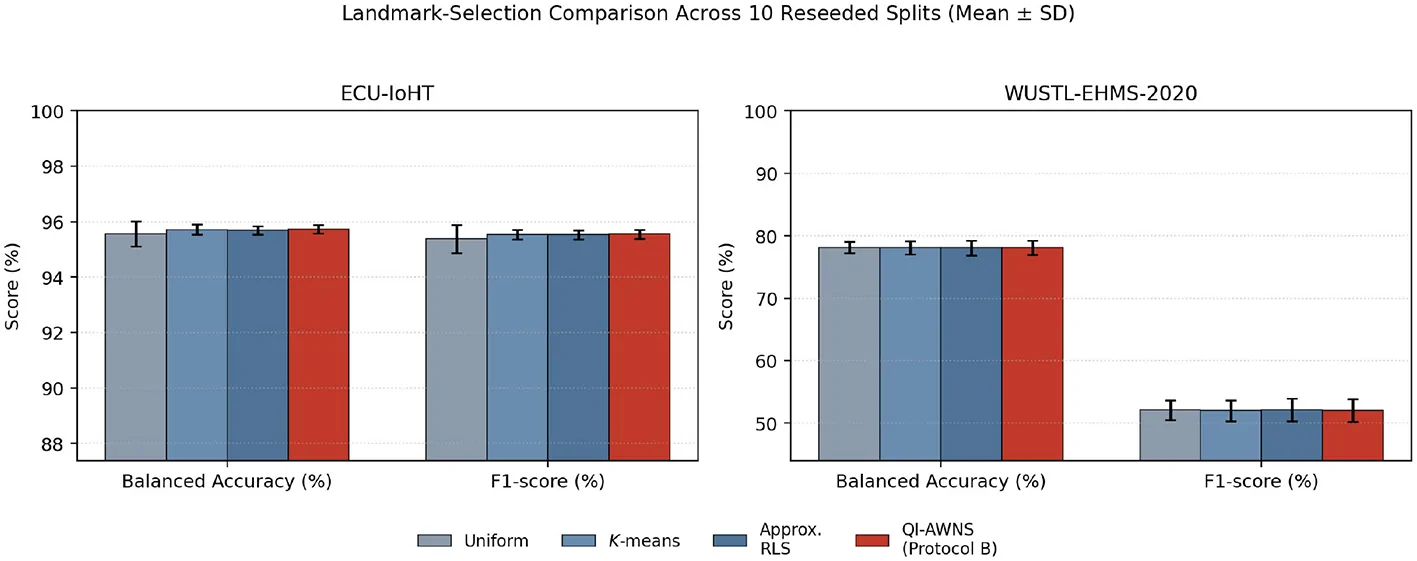
Landmark-selection method comparison across 10 reseeded splits (mean ± 1 SD), ECU-IoHT and WUSTL-EHMS-2020. QI-AWNS is shown under Protocol B (per-seed returned weights, Section 4.3), its fairer-evaluated configuration. Overlapping error bars illustrate directly why none of the pairwise differences in Table 11 survive multiple-comparison correction.

#### 4.3.1 Ablation study and importance-weight search

Table 13 reports the 8-variant ablation (Section 3.6) at seed 42. On ECU-IoHT, the density-only (representativeness) variant matches the full QI-AWNS result exactly, because the validation-tuned weight search (Table 9) selected λ_*r*_ = 1 (pure representativeness) as the best-scoring combination on this dataset; uncertainty-only and boundary-only variants are the two clearly *worse*-performing single components (accuracy 90.94–90.99% vs. 93.09% uniform), showing that not every importance signal helps, and some actively hurt, on this dataset. The deterministic top-*m* variant (no probabilistic sampling, Section 3.6) performs within the noise of the full stochastic QI-AWNS on both datasets, offering no evidence that Born-rule-style probabilistic sampling itself, as opposed to the underlying importance score, contributes measurably here.

**Table 13:** 8-variant ablation, single split (seed 42).

Dataset	Variant	Acc. (%)	Bal. Acc. (%)	Rec.	F1	AUC	MCC
ECU-IoHT	Uniform (no scoring)	93.09	95.62	91.25	95.42	0.9902	0.829
	Uncertainty-only	90.99	94.29	88.58	93.95	0.9728	0.788
	Boundary-only	90.94	94.18	88.58	93.92	0.9730	0.786
	Density-only	93.18	95.68	91.36	95.48	0.9911	0.831
	Minority-only	92.99	95.30	91.31	95.36	0.9863	0.825
	Full QI-AWNS (tuned weights)	**93.18**	**95.68**	91.36	95.48	0.9911	0.831
	Full, deterministic top-*m*	93.12	95.64	91.28	95.44	0.9918	0.830
	Full, equal weights	93.03	95.23	91.42	95.39	0.9911	0.825
WUSTL	Uniform (no scoring)	84.19	79.10	72.31	53.43	0.8884	0.470
	Uncertainty-only	84.15	78.94	71.99	53.25	0.8875	0.468
	Boundary-only	84.15	79.22	72.64	53.48	0.8890	0.471
	Density-only	84.03	79.43	73.29	53.51	0.8883	0.472
	Minority-only	84.07	79.45	73.29	53.57	0.8889	0.473
	Full QI-AWNS (tuned weights)	84.11	78.64	71.34	52.96	0.8883	0.464
	Full, deterministic top-*m*	84.15	79.36	72.96	53.59	0.8875	0.473
	Full, equal weights	84.15	**79.92**	**74.27**	**54.03**	0.8893	**0.479**

For ECU-IoHT, bold indicates the accuracy and balanced-accuracy values of the Full QI-AWNS configuration, the study's validation-selected and formally reported result, which ties the best outcome among all eight variants on this dataset. For WUSTL-EHMS-2020, bold indicates the highest value achieved in each column among all eight variants, corresponding to the equal-weights variant discussed in Section 4.3 as an instance of validation-set ranking reversal.

The most instructive single result in Table 13 is on WUSTL-EHMS-2020: the *equal-weighted* variant (λ_*u*_ = λ_*b*_ = λ_*r*_ = λ_*m*_ = 0.25) *was* one of the 10 candidates scored during the validation-only weight search (Table 9), where it reached 75.54% validation balanced accuracy, lower than the selected candidate (λ_*u*_, λ_*b*_, λ_*r*_, λ_*m*_) = (0.5, 0.3, 0.1, 0.1)'s 76.26%, which is why the search chose the latter. On the held-out test set, however, this ranking *reverses*: equal weights reaches 79.92% balanced accuracy and 74.27% recall, clearly exceeding the validation-selected configuration's 78.64% balanced accuracy and 71.34% recall (Table 13). This is not a case of an untested configuration outperforming a tested one; it is a direct demonstration that a small (2,448-sample), 12.5%-minority-class validation partition can rank two *already-evaluated* candidates in the wrong order relative to test: the validation signal that favored (0.5, 0.3, 0.1, 0.1) over equal weights by 0.72 percentage points did not generalize, and test reversed the ordering by a substantially larger 1.28-point margin. We emphasize that this observation is a *post-hoc* diagnostic, not a model-selection step: QI-AWNS's reported configuration on WUSTL-EHMS-2020 remains the validation-selected weight vector (0.5, 0.3, 0.1, 0.1) throughout every other table and figure in this paper; we did not retroactively substitute the equal-weighted variant, re-score it as “the” QI-AWNS result, or use this test-set comparison to choose between the two candidates. The single held-out test evaluation specified in Section 3.2 is used exactly once for that purpose elsewhere in this paper; here it is instead used, after the fact and for both ablation candidates simultaneously, purely to illustrate a validation-set reliability risk, and does not feed back into any reported accuracy claim, weight selection, or deployment recommendation.

Figure 9 visualizes Table 13's balanced-accuracy column as each variant's change relative to the uniform-sampling baseline, in percentage points, on a single shared axis for both datasets, so each component's individual contribution (and, on WUSTL-EHMS-2020, the equal-weights reversal discussed above) is directly comparable across datasets rather than only visible within each dataset's own raw accuracy range (an earlier version of this figure plotted raw balanced accuracy on two panels with different y-axis starting points, 92% and 76%, which made cross-dataset comparison harder than necessary).

**Figure 9:**
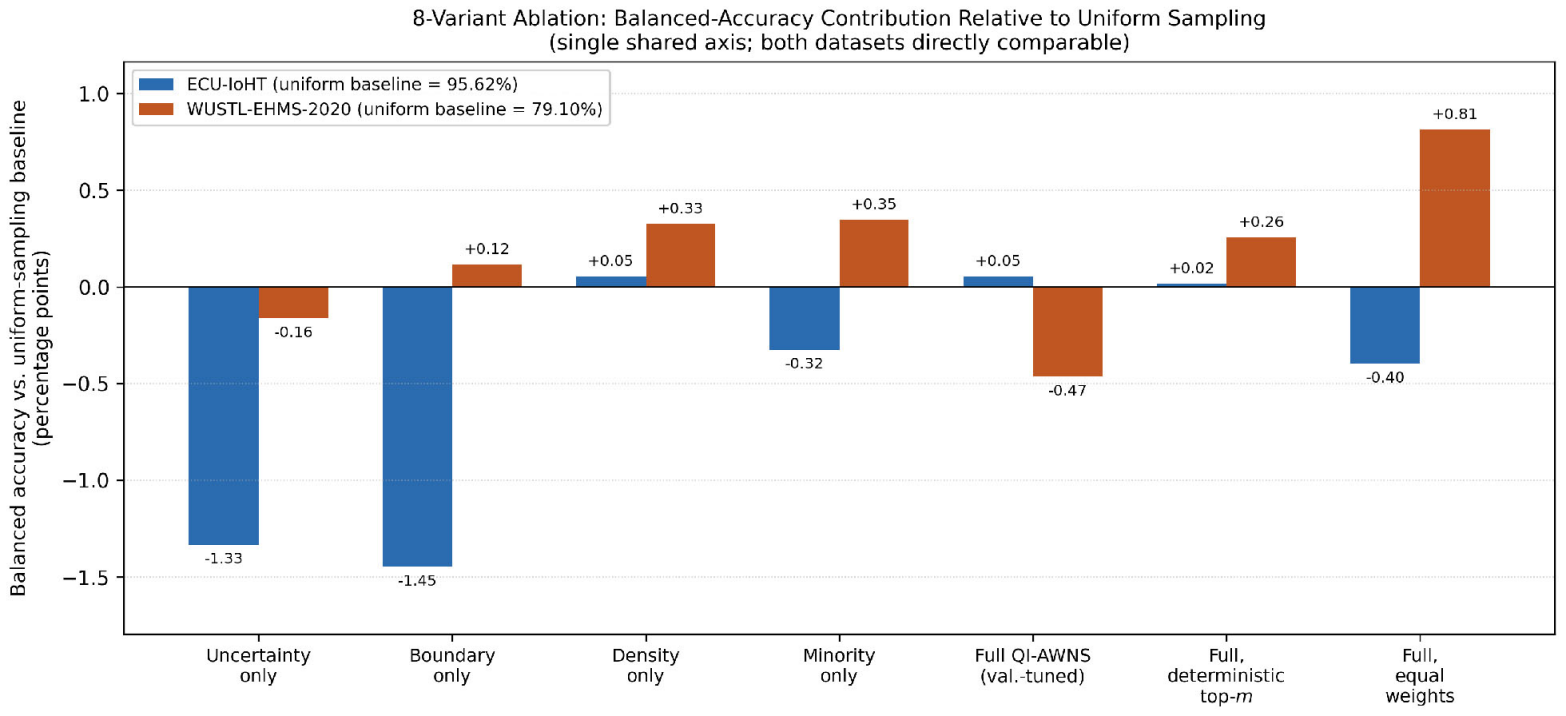
Ablation-contribution plot: each of the 7 non-uniform QI-AWNS variants' balanced-accuracy change relative to the uniform-sampling baseline (seed 42), in percentage points, ECU-IoHT and WUSTL-EHMS-2020 on one shared axis. The validation-tuned full configuration (“Full QI-AWNS”) is the only variant that underperforms uniform sampling on WUSTL-EHMS-2020, at −0.47 points–the equal-weights reversal finding discussed in Section 4.3. No individual component or variant exceeds ±1.5 points on either dataset.

Table 9 makes the equal-weights finding precise: on WUSTL-EHMS-2020, equal weights (row 5, 0.7554) scored below the selected candidate (row 8, (0.5, 0.3, 0.1, 0.1), 0.7626) on validation, a 0.72-point gap that reverses to a 1.28-point gap in the *opposite* direction on the held-out test set (Table 13). On ECU-IoHT, two candidates tie for the best validation score (density-only, and minority-heavy (0.1, 0.1, 0.4, 0.4), both 0.9576); we broke the tie by selecting the first-encountered candidate (density-only), which Table 13 shows also ties for the best test-set result on that dataset.

### 4.4 Feature selection analysis

Figures 1, 10 show the real, code-computed ANOVA F-statistic ranking used to select features for both datasets (Section 3.3). On WUSTL-EHMS-2020, 7 of the 37 raw features (Dport, SrcGap, DstGap, DIntPktAct, dMaxPktSz, dMinPktSz, Trans) are constant within the training partition and are treated as *F* = 0 for ranking purposes (Section 3.3); we surface this explicitly in the figure rather than silently omit the affected columns.

**Figure 10:**
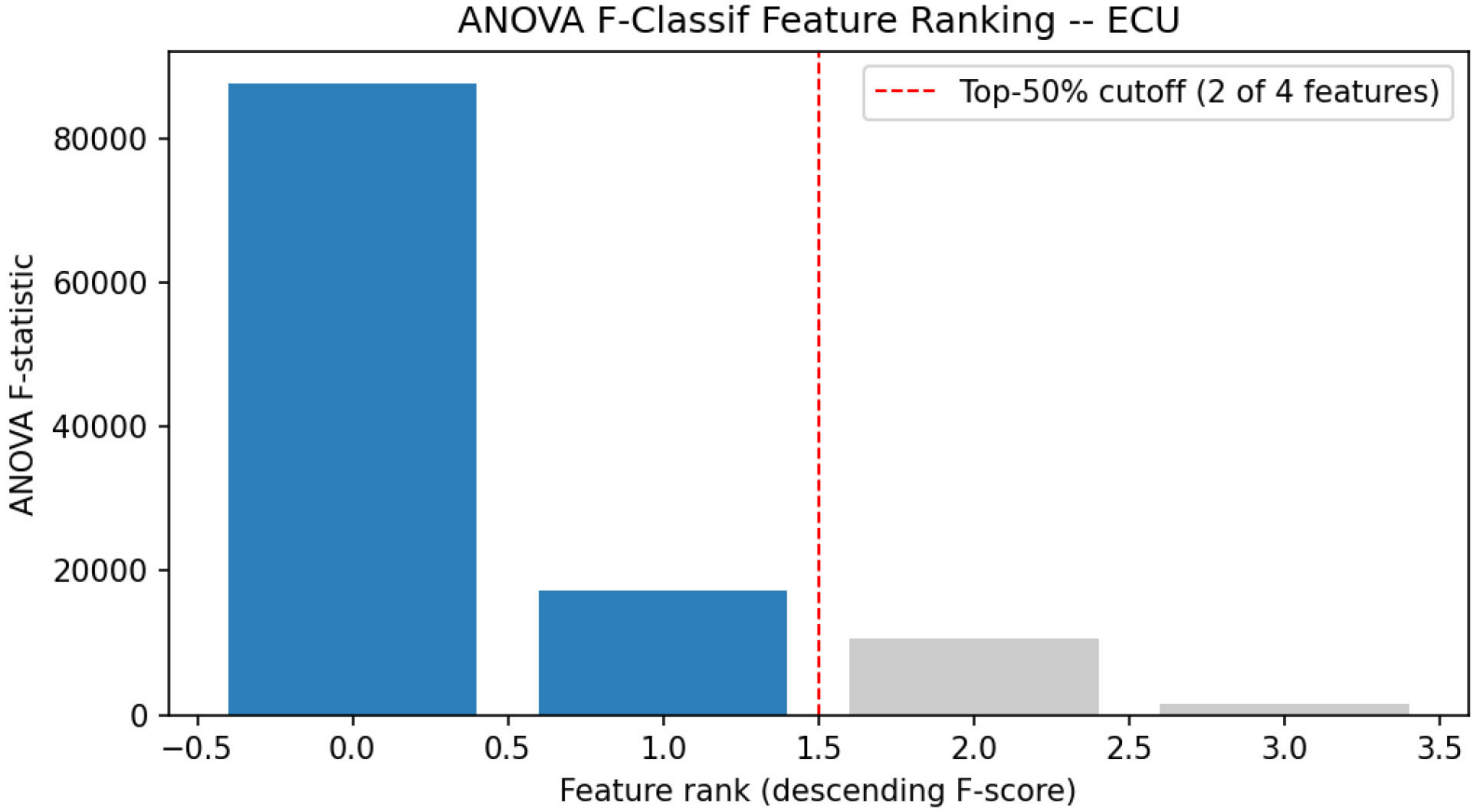
ANOVA F-statistic feature ranking, ECU-IoHT (4 raw features; top-50% selection cutoff marked).

### 4.5 Computational cost

Table 14 summarizes fit time across configurations. The *a priori* configuration is inexpensive on both datasets (under 16 seconds), and even the full matched-search cost (30 evaluations plus final refit) remains under two minutes on ECU-IoHT and under 40 seconds on WUSTL-EHMS-2020, costs we consider practical for periodic gateway-side retraining, though we did not measure inference-time latency or memory footprint on actual edge hardware (Section 6).

**Table 14:** Total fit/search time by configuration.

Configuration	ECU-IoHT (s)	WUSTL-EHMS-2020 (s)
A priori (single fit)	15.69	1.07
Matched search (30 evals)	105.9	38.4
Matched search, final refit	14.3	0.11
Matched search, total	120.2	38.5

Table 3 extends this to the four landmark-selection strategies (Section 3.6, seed 42): fit time, per-sample inference latency, derived throughput (1000/latency), serialized model size (landmarks plus classifier coefficients, pickled), and peak Python-level memory during fit (tracemalloc, not total process RSS). Across datasets, all four strategies are within a small constant factor of one another on every efficiency axis; landmark-selection strategy affects predictive metrics (Table 8) far more than computational cost, since the downstream Nyström transform and classifier are identical regardless of which points were chosen as landmarks. Model size scales with the embedding dimension *m* and the raw feature count per dataset (80.8 KB at ECU-IoHT's *m* = 100 vs. 2.90 MB at WUSTL-EHMS-2020's *m* = 600), not with landmark-selection strategy. *This bears directly on this paper's “lightweight” and “edge” framing*: every figure in Tables 3, 14 was measured on this sandbox's commodity CPU, not on a Raspberry Pi, microcontroller, or other representative edge-hardware target; these numbers reflect relative cost among the compared methods on one shared, non-edge machine, not edge-deployment performance (Section 6).

Figure 11 visualizes Tables 3, 14 together: the left panel shows that landmark-selection fit time is a small, comparable cost across all four strategies on both datasets, while the right panel shows the much larger total-pipeline-cost gap between the *a priori* and matched-search configurations (log scale), making it clear that landmark-selection strategy, not the matched hyperparameter search, is the negligible cost component here.

**Figure 11:**
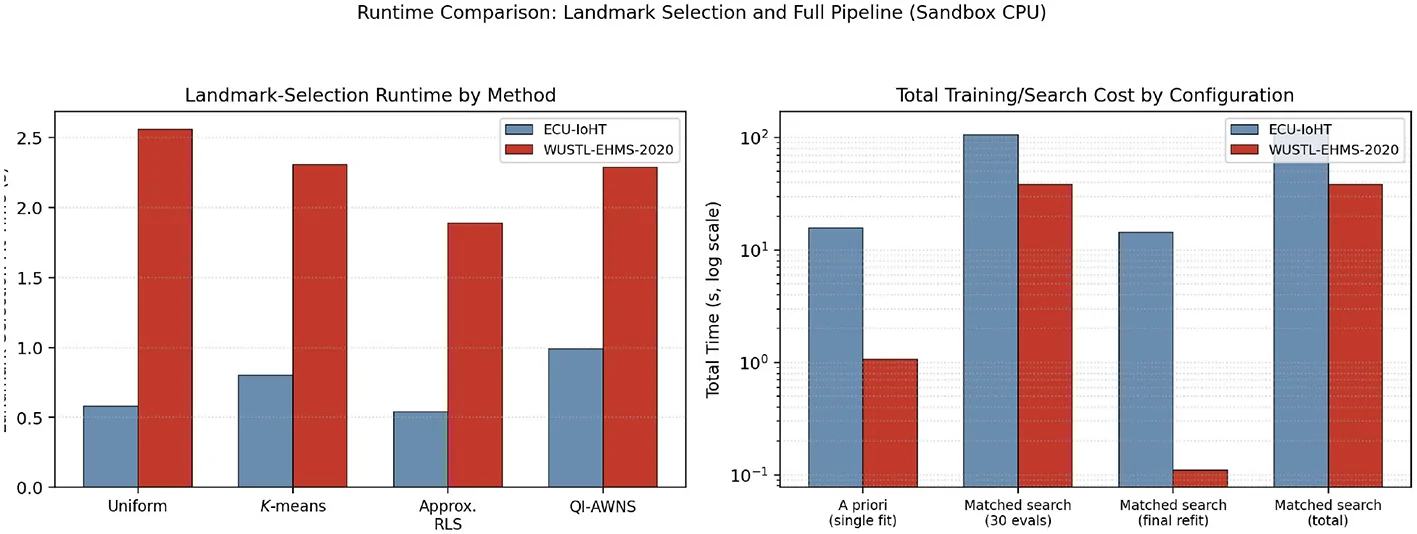
Runtime comparison: landmark-selection fit time by method (**left**) and total training/search cost by configuration (**right**, log scale), both datasets, sandbox CPU (Section 6).

## 5 Discussion

This study directly addresses the concerns about QI-AWNS, and we report the mixed, protocol-dependent results in full: under frozen importance weights (Protocol A), QI-AWNS is statistically indistinguishable from, or nominally worse than, uniform, *K*-means, and approximate ridge-leverage-score Nyström landmark selection across 10 reseeded splits on both datasets (Table 11). Its single-split McNemar advantage over uniform sampling on ECU-IoHT (*p* = 1.2 × 10^−4^) does not survive reseeding, as two of ten reseeds show a clear regression (Table 10). Retuning the importance weights independently within each reseed's own validation split (Protocol B) recovers these regressions and yields a small, numerically favorable difference over uniform sampling specifically on ECU-IoHT (mean paired difference +0.18 points in balanced accuracy, Table 12; raw Wilcoxon *p* = 0.022, Holm-corrected *p* = 0.129; Protocol B vs. A directly, *p* = 0.016); this effect does not survive correction for the family of six paired comparisons run under each protocol. QI-AWNS still does not demonstrate an advantage over the two established non-uniform baselines (*K*-means, approximate ridge-leverage-score) on either dataset, or any advantage at all on WUSTL-EHMS-2020, under either protocol. Giving both the approximate ridge-leverage-score and *K*-means baselines their own validation-tuned free parameters (Section 6, item 4) only widens this gap on WUSTL-EHMS-2020 (tuned leverage 79.38%, tuned *K*-means 79.43%, both above QI-AWNS's 78.64%), so QI-AWNS's null result is not an artifact of comparing a tuned method against under-tuned baselines. The ablation and weight-search results (Section 4.3) reinforce why both protocols matter: on WUSTL-EHMS-2020, an equal-weighted combination of QI-AWNS's four components, one of the 10 candidates the validation-only search evaluated and scored lower than the eventual winner, reverses that ranking and outperforms the validation-selected configuration on the held-out test set, showing that a single seed's validation-based ranking of two already-evaluated candidates did not generalize to test. We read this as evidence that (i) evaluating the complete model-selection procedure across repeated splits, not just the final selected weights, is required to characterize an importance-weighted sampling method fairly (an evaluation-methodology claim, not that QI-AWNS is unstable in deployment, since a deployed system can simply ship the retuned weights); and (ii) even under the fairer, retuned protocol, QI-AWNS's difference from the weakest (uniform) baseline is small and does not survive multiple-comparison correction, so we do not claim a broad improvement over the landmark-selection state of the art.

**An indirect implication of this negative result**. Both datasets in this study already select landmarks at *m* in the hundreds (*m* = 100 for ECU-IoHT, *m* = 600 for WUSTL-EHMS-2020, Table 3). At this scale, none of the three non-uniform strategies–*K*-means, approximate ridge-leverage-score sampling, or QI-AWNS–produced a statistically robust advantage over plain uniform sampling on either dataset after multiple-comparison correction. We did not sweep *m* within a single dataset to test this directly, so we state it as a hypothesis consistent with our results rather than a demonstrated scaling law. It is consistent with a broader pattern in the Nyström approximation literature: landmark-selection strategy matters most when *m* is small relative to the kernel matrix's effective rank, and its influence should shrink as *m* grows large enough that even a uniformly random landmark set adequately spans the relevant subspace. If this holds more generally, the practical implication is that once a practitioner is already budgeting landmarks in the hundreds, further investment in landmark-selection sophistication may be better redirected elsewhere–feature engineering, kernel choice, or classifier tuning–than in an increasingly intricate landmark-selection heuristic. We list a controlled sweep of *m* across a wider range, holding the dataset and landmark strategy fixed, as concrete future work to test this hypothesis directly (Section 7).

Separately from the landmark-selection question, the underlying Nyström/random-feature model's raw accuracy is not uniformly ahead of simple tabular baselines. On ECU-IoHT, a matched-effort hyperparameter search substantially improves the *a priori* classifier configuration (93.27% → 96.39%), yet three of five simple tabular baselines (random forest, XGBoost, the MLP) still exceed even this matched-search result with unsearched, conventional configurations. On WUSTL-EHMS-2020, a matched-effort search actively *hurts* the model (92.89% → 87.95%), and two baselines (random forest, MLP) match or exceed its best *a priori* result. No dataset in this study shows the Nyström/random-feature construction's raw accuracy clearly exceeding the best tabular baseline; its practical case rests on training cost and model compactness (Table 14; embedding dimensions of 100–600 vs., e.g., a random forest's 200 trees or an MLP's tens of thousands of weights), not on an accuracy advantage.

We consider two plausible, non-exclusive explanations for the WUSTL-EHMS-2020 matched-search regression, without claiming to have confirmed either. First, our random search selects candidates based on raw validation accuracy on a dataset in which the attack class comprises only 12.5% of samples. As a result, a search step can trade off minority-class recall for a small accuracy gain, unlike the hand-set *a priori* configuration; widening the search space to include classifier family did not close this regression, since the search still selected LinearSVC as its best WUSTL-EHMS-2020 candidate. Second, WUSTL-EHMS-2020's training partition (11,422 samples) is roughly seven times smaller than ECU-IoHT's (77,844 samples), and small training partitions are known to disadvantage aggressive hyperparameter search more broadly (Grinsztajn et al., 2022). We did not run the ablation needed to separate these explanations (e.g., scoring the search on balanced accuracy or F1) and list it as required follow-up (Section 6).

**Why ECU-IoHT and WUSTL-EHMS-2020 behave so differently, more broadly**. The matched-search regression above is one symptom of a wider pattern: nearly every result in this paper is more favorable, or at least more stable, on ECU-IoHT than on WUSTL-EHMS-2020, which we attribute to four compounding structural differences rather than any single cause. First, class balance: ECU-IoHT's test partition is attack-majority, whereas WUSTL-EHMS-2020's attack class is only 12.5% of the test partition (Section 3.1). A search scored on raw accuracy (Section 3.8) has far more room to overfit the majority class without that overfitting being visible in the scoring metric. Second, training-partition size differs roughly sevenfold (77,844 vs. 11,422 samples), and smaller training partitions are known to make model fitting and hyperparameter search noisier (Grinsztajn et al., 2022), compounding the class-imbalance point since WUSTL-EHMS-2020's smaller partition also has fewer positive-class examples in absolute terms. Third, the datasets differ sharply in raw feature richness: ECU-IoHT exposes only 4 raw features (2 selected, a genuine data limitation, Section 6), while WUSTL-EHMS-2020 exposes 37 raw features (18 selected) spanning biometric and network-flow modalities, with a correspondingly larger embedding dimension (*m* = 100 vs. *m* = 600, Table 3) that is intrinsically harder for any landmark-selection heuristic to summarize well with a fixed budget. Fourth, QI-AWNS's importance-weight search selected a single dominant component on ECU-IoHT (pure representativeness, λ_*r*_ = 1) but a diffuse four-component blend on WUSTL-EHMS-2020 (λ_*u*_ = 0.5, λ_*b*_ = 0.3, λ_*r*_ = 0.1, λ_*m*_ = 0.1); a search converging on one clearly-best component is inherently less sensitive to which validation split it saw, consistent with why Protocol B's retuning stabilized ECU-IoHT but not WUSTL-EHMS-2020. We present these four factors as the most plausible structural account available, not a confirmed causal explanation; disentangling their individual contributions is listed as future work (Section 7).

Returning to the gap relative to prior literature (Section 2): our leakage-free WUSTL-EHMS-2020 accuracy (92.89% in the best case) is markedly lower than the ~94–99.9% range reported by several prior studies for the same public dataset (Tauqeer et al., 2022; Judith et al., 2023; Shaikh et al., 2024; Aversano et al., 2026; Abdelhaq et al., 2026; Krishna Kumari et al., 2026). A plausible partial explanation is our leakage-free protocol rather than a weaker method, since none of the cited prior studies' published descriptions document split-before-fit feature selection and scaling to the level of detail provided in Section 3.2. We emphasize that this reflects differences in what each paper's published description allows us to verify, not a demonstrated finding that those studies' higher numbers were caused by leakage; we have not directly reproduced their pipelines, and their results may well be genuine. The same caution applies to ECU-IoHT, where several prior studies report accuracies above 99% (Vijayakumar et al., 2023; Algethami and Alshamrani, 2024; Alohali et al., 2025; Krishna Kumari et al., 2026) against our best (matched-search) result of 96.39%.

Tables 1, 2 (Section 2) already list each of these deep/complex-model results and their reported accuracies alongside our own leakage-free numbers (96.39% ECU-IoHT matched-search, 92.89% WUSTL-EHMS-2020 *a priori*), so we do not repeat the per-study figures here. **These reported results cannot be compared directly because previous studies did not use a verified split-before-fit evaluation protocol**; this is precisely why those tables' “Split-Before-Fit Protocol Documented?” column is included, to keep that qualification visible in the tables themselves, not only in surrounding prose.

Figure 12 places these numbers side by side rather than leaving the comparison only in running text. On both datasets, this paper's leakage-free results sit visibly below the cluster of prior-reported accuracies (a gap of roughly 3–7 points on ECU-IoHT and 1–7 points on WUSTL-EHMS-2020, depending on which prior study is the comparison point); the figure deliberately omits which explanation, our weaker configuration or their possible leakage, is correct, since we cannot confirm attribution without reproducing the cited studies. The figure's top row also places the five tabular baselines (Section 3.9) alongside both the RFK model and the same prior-literature figures, so the tabular baselines' competitiveness (Section 5 above) is visible directly against the literature; its bottom row reports balanced accuracy and MCC, two metrics more informative than raw accuracy under class imbalance (Section 3.1), for our own methods only, since the prior-literature studies cited in Sections 2.3 and 2.4 did not, to our knowledge, publish those metrics. We did not identify a Transformer-based or graph-neural-network intrusion-detection study evaluated specifically on ECU-IoHT or WUSTL-EHMS-2020, which is why no such entry appears in Figure 12; this gap motivates the broader model-class comparison listed as future work (Section 7).

**Figure 12:**
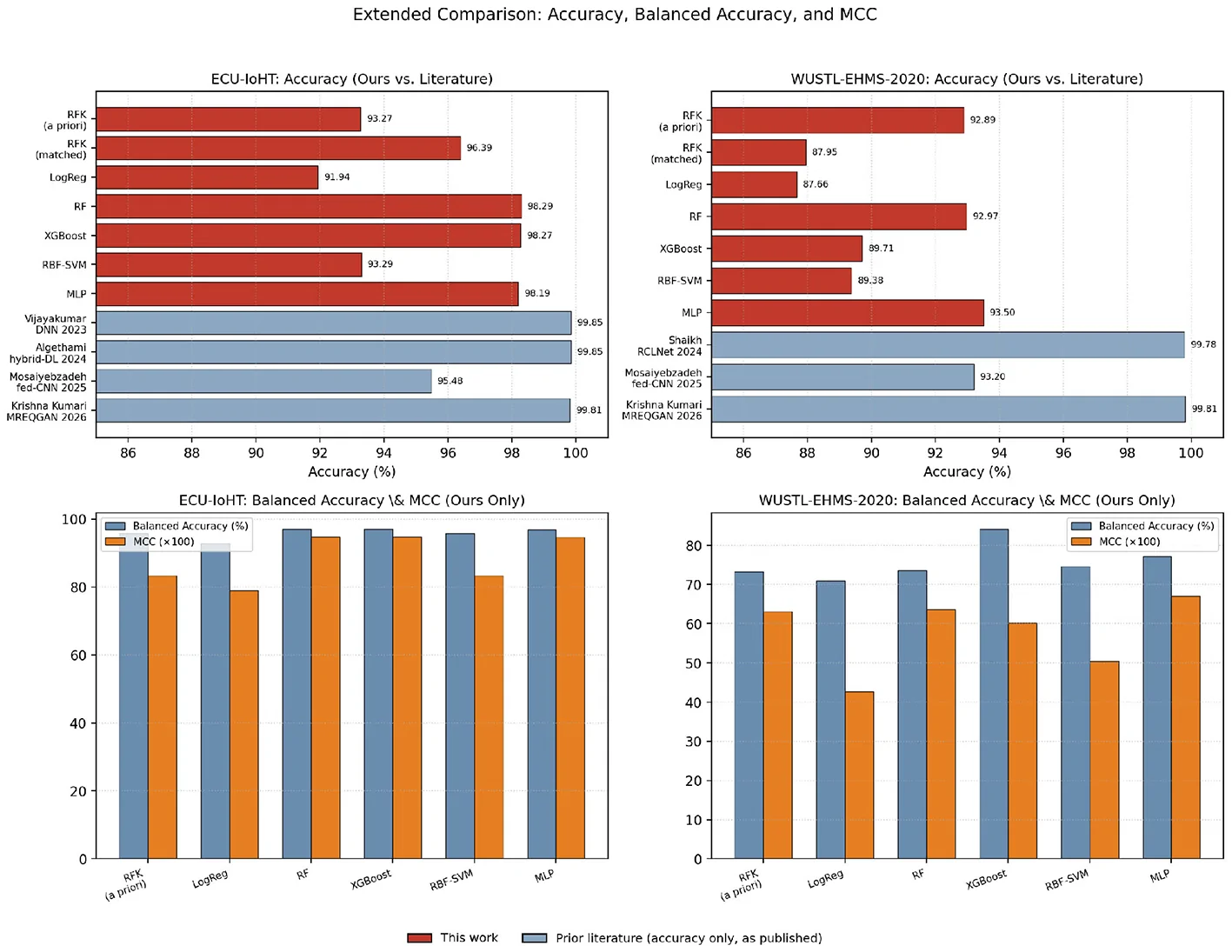
Comparison with prior literature and tabular baselines. Top: accuracy, this work (RFK and five tabular baselines) vs. prior literature, both datasets. Bottom: balanced accuracy and MCC for this work's methods only (not reported by the cited prior studies, so not plotted for them). This comparison is not a controlled, like-for-like test: the ECU-IoHT panel in particular compares our results, obtained on a 4-raw-feature version of the dataset, against prior studies that, in some cases, used richer feature sets, so the visible gap should not be read as attributable to leakage alone (Table 1's note; Section 5).

From a deployment perspective, the practical argument for this class of model is the one Table 14 makes directly: a sub-two-minute total training cost, including a full 30-evaluation hyperparameter search, on commodity hardware, vs. the far larger memory and compute footprints typically associated with the deep and multi-component architectures reviewed in Section 2. We recommend that IoMT IDS studies proposing this model class report cost on a comparable, total-time basis and against simple tabular baselines as a matter of course, rather than reporting accuracy alone against other complex architectures.

**Where QI-AWNS's practical value lies, given that its accuracy advantage is small and largely uncorrected**. Because Section 4.3's headline accuracy comparison is a near-null result, it is worth stating plainly what QI-AWNS still offers. First, *interpretability of the selection mechanism*: unlike *K*-means (synthetic centroids with no direct semantic meaning) or approximate ridge-leverage-score sampling (a matrix-algebraic quantity without an operational reading), QI-AWNS's four components are individually named and inspectable; Table 9's per-dataset selected weights already function as a diagnostic, since the search's convergence on pure representativeness for ECU-IoHT vs. a diffuse blend for WUSTL-EHMS-2020 is itself an interpretable signal about which sampling criterion each dataset's decision problem rewards. Second, *stability under the correct evaluation protocol*: Protocol A's frozen weights showed real, reseed-dependent regressions (two of ten seeds on ECU-IoHT), and Protocol B's per-seed retuning recovers a procedure that no longer regresses, telling a practitioner that QI-AWNS should be deployed with its retuning step intact. Third, *efficiency is not compromised*: Section 3.6.1 and Table 3 together show that QI-AWNS's additional *O*(|pool|^2^*d*) nearest-neighbor term costs well under a second of extra fit time at the pool sizes used here, with no measurable inference-latency or model-size penalty relative to the three baselines. We present these–interpretability, protocol-dependent stability, and negligible added cost–as the honest basis for QI-AWNS's practical value, rather than restate an accuracy claim the corrected statistics do not support.

Returning to the patient-safety framing of Section 1: on WUSTL-EHMS-2020, the RFK model's best (*a priori*) attack-class recall is 46.91%, meaning it misses more than half of true attacks in our test partition. This would not, on its own, be a reassuring detection rate for a system whose stated motivation is to protect patient safety. We do not consider it evidence that this model, or any model compared here, is deployment-ready as-is on this dataset. We return to this point in the Conclusion's future-work list rather than understating it here.

## 6 Limitations

1. **Candidate-pool restriction for landmark selection**. All four landmark-selection strategies (Section 3.6) draw from a stratified random candidate pool of size min(5000, *n*_train_) rather than the full training partition, for computational tractability; this restriction is applied identically across strategies, so it does not advantage any one method, but it means none of the four strategies was tested at the full training-set candidate scale.
2. **Frozen-weight evaluation alone would have been misleading; Protocol B still has its own scope limits**. An earlier version of this evaluation used only frozen (seed-42-tuned) weights (Protocol A) and concluded that QI-AWNS showed no advantage anywhere. Adding Protocol B (independent per-seed retuning) overturned that conclusion specifically on ECU-IoHT, so any single-protocol evaluation of an importance-weighted sampling method should be treated cautiously. We did not test a third alternative (e.g., nested cross-validation for weight selection within each seed).
3. **The importance-weight grid was small (10 candidates)** and did not include intermediate points between the tested vectors in either protocol; a finer or Bayesian-optimization-based search might select different, possibly more robust, weightings. Section 4.3's finding that a single seed's validation-based ranking of two already-evaluated candidates reversed on WUSTL-EHMS-2020's test set suggests that a finer search alone would not fix the underlying risk of small-validation-set overfitting there, though Protocol B's retuning did resolve an analogous instability on ECU-IoHT. **This test-set comparison is an exploratory**, ***post-hoc* diagnostic of validation-set reliability, not a re-run model-selection step**: it does not change QI-AWNS's reported, validation-selected configuration; a fully nested repeated-evaluation design that re-tunes and re-tests across many splits, rather than the single held-out test partition specified in Section 3.2, is listed as future study (Section 7).
4. **Baseline tuning asymmetry, closed**. An earlier version of this comparison left both the approximate ridge-leverage-score baseline's regularization multiplier and mini-batch *K*-means' initialization count fixed at default values (reg_mult = 0.1 in lam = trace(*K*)/*n*·reg_mult+10^−6^; n_init = 3) while QI-AWNS's importance weights were validation-tuned (Section 4.3). We closed this asymmetry for both baselines with matched 10-point validation-only grid searches (matching the size of QI-AWNS's own weight grid). For the leverage baseline, the search selected reg_mult = 0.001 on ECU-IoHT, where the swept range changed validation balanced accuracy by less than 0.2 points and the tuned test result (95.64%) was numerically identical to the untuned default; on WUSTL-EHMS-2020 it selected reg_mult = 0.03, raising test balanced accuracy from 79.06% (untuned) to **79.38%**. For *K*-means, the search over n_init selected n_init = 1 on ECU-IoHT, raising test balanced accuracy from 95.64% (untuned default) to **95.70%**; on WUSTL-EHMS-2020 it selected n_init = 2, raising test balanced accuracy from 78.80% (untuned) to **79.43%** – the highest of all four landmark-selection strategies on that dataset (uniform 79.10%, tuned leverage 79.38%, QI-AWNS 78.64%). Uniform sampling has no tunable free parameter, so no equivalent search applies to it. Giving every non-uniform baseline an equivalent validation-tuning pass strengthens, rather than undermines, this paper's central finding: on WUSTL-EHMS-2020, two of the three classical alternatives now outperform QI-AWNS once tuned equivalently, so QI-AWNS's lack of demonstrated advantage is not an artifact of an under-tuned comparison.
5. **No physical-hardware deployment measurement**. All training-cost and inference-latency figures were measured on commodity server/cloud CPUs, not on the low-power gateways or embedded microcontrollers (e.g., Raspberry Pi, ESP32, embedded FPGA) that this paper's motivation targets. We list physical-hardware validation as required, not optional, future work (Section 7).
6. **Baseline hyperparameters were not tuned**. The five tabular baselines (Section 3.9) use conventional, fixed hyperparameters rather than a matched validation-only search; a better-tuned baseline could plausibly widen the gaps reported in Section 4.
7. **The WUSTL-EHMS-2020 matched-search regression (Section** **4.2) is not fully explained**. Section 5 offers two plausible but unconfirmed accounts; we did not implement or test a balanced-accuracy-scored version of that specific hyperparameter search (as distinct from the landmark-selection weight search, which we did score using balanced accuracy).
8. **ECU-IoHT feature-count gap vs. prior literature**. The file available to us contains 4 raw features (2 selected), substantially fewer than the original ECU-IoHT publication's feature set (Section 3.1).
9. **No adversarial-robustness or concept-drift evaluation**, both standard concerns for any IDS proposed for long-running hospital deployment (Goodfellow et al., 2015; Gama et al., 2014).
10. **The literature review (Section** **2) is a narrative, per-dataset review, not a formal systematic review**: it does not follow a predeclared search string, database list, or inclusion/exclusion criteria. Therefore, statements about what the reviewed literature does or does not report (e.g., Section 2.5) describe the studies we identified and discuss, not an exhaustively verified claim about the entire published literature.
11. **We have not directly reproduced the prior studies cited in Section** **2** **to confirm our leakage-based attribution of the accuracy gap against their reported numbers (Section** **5); this attribution remains plausible, not demonstrated**.

## 7 Conclusion and future work

We proposed a task-aware, importance-weighted Nyström landmark-selection mechanism with an optional quantum-amplitude reparameterization (QI-AWNS) that we showed is mathematically equivalent to direct score-proportional normalization (Section 3.6). Thus, the genuine contribution is the four-component importance scoring and its rigorous evaluation, not the amplitude framing. We evaluated it, including its mixed, protocol-dependent result, against uniform, *K*-means, and approximate ridge-leverage-score landmark selection, five tabular baselines, and a full-kernel RBF-SVM, under a leakage-free, split-before-fit protocol on two IoMT intrusion-detection benchmarks. Across 10 reseeded splits, a frozen-weight configuration (Protocol A) showed no advantage over any baseline on either dataset; retuning weights per reseed (Protocol B) recovered ECU-IoHT's frozen-weight regressions and produced a small, uncorrected-only difference over uniform sampling on that dataset (mean +0.18 points balanced accuracy; raw *p* = 0.022, Holm-adjusted *p* = 0.129), with no advantage over *K*-means or approximate ridge-leverage-score sampling on either dataset. An 8-variant ablation and weight search further showed that a single seed's validation-based ranking of two already-evaluated weight candidates reversed on WUSTL-EHMS-2020's test set, demonstrating why evaluating the complete model-selection procedure across repeated splits, not a one-time validation search, is required to characterize this class of method fairly. Separately, the underlying Nyström/random-feature (RFK) model's raw accuracy (93.27% ECU-IoHT, 92.89% WUSTL-EHMS-2020 *a priori*) is matched or exceeded by simple tabular baselines on both datasets using unsearched configurations, so this paper's practical case rests on training cost and model compactness, not accuracy.

We consider it more valuable to report QI-AWNS's null result carefully than to selectively report only the single favorable single-split *p*-value. Five directions for future work follow directly from Section 6:

1. **Physical-hardware validation**. Deploying and measuring this pipeline directly on low-power hardware (e.g., Raspberry Pi, ESP32-class microcontrollers, or an embedded FPGA) to obtain real inference latency and memory-footprint figures, rather than proxy measurements on commodity server hardware.
2. **Larger-scale, finer-grained QI-AWNS weight search, fully nested against the test partition**. This paper's per-seed retuning (Protocol B, Section 4.3) already shows that re-tuning importance weights independently at each reseed materially changes the ECU-IoHT result; remaining work is to remove the min(5000, *n*_train_) candidate-pool restriction, test a finer or Bayesian-optimization-based search over the weight simplex, and formalize Section 6's exploratory test-set diagnostic as a properly nested repeated-evaluation design (re-tuning and re-testing across many independent splits) to determine whether a more thorough, test-set-blind search would also close the gap against *K*-means and approximate ridge-leverage-score sampling, or against any baseline on WUSTL-EHMS-2020.
3. **Balanced-accuracy-scored version of the main hyperparameter search**. Directly testing whether scoring the Section 3.8 matched-budget search (as distinct from the landmark-weight search already balanced-accuracy-scored in Section 4.3) on balanced accuracy or F1 resolves the WUSTL-EHMS-2020 regression identified in Section 5.
4. **Broader model-class and dataset-scope comparison**. This paper deliberately restricts its comparison set to a full-kernel RBF-SVM and five simple tabular baselines (Section 3.9), chosen to test whether the Nyström/random-feature construction earns its complexity relative to conventional, lightweight alternatives; it does not include a Transformer-based or graph-neural-network (GNN) IDS architecture, both increasingly common in the broader IoMT/IoT intrusion-detection literature (Kalakoti et al., 2024; Zhong et al., 2024), though we did not find one evaluated on ECU-IoHT or WUSTL-EHMS-2020 specifically (Section 2). Adding such a comparison would test whether QI-AWNS's efficiency and interpretability case (Section 5) still holds against architectures designed for higher raw accuracy at greater computational cost, rather than against other lightweight baselines. Separately, evaluating this pipeline on a third, genuinely leakage-free IoMT benchmark would test whether the cross-dataset differences identified in Section 5 (class balance, training-set size, feature dimensionality) generalize as structural explanations, or are specific to ECU-IoHT and WUSTL-EHMS-2020.
5. **A controlled sweep of landmark count** **m**. Both datasets in this study fix *m* to a single per-dataset value (100 for ECU-IoHT, 600 for WUSTL-EHMS-2020); Section 5 notes that the landmark-selection strategy showed no robust advantage at either of these already-hundreds-scale values, consistent with (but not a direct test of) the hypothesis that strategy matters less as *m* grows relative to the kernel matrix's effective rank. Holding one dataset fixed and comparing uniform, *K*-means, approximate ridge-leverage-score, and QI-AWNS sampling across a swept range of *m* (e.g., from tens to the low thousands) would test this directly and clarify whether landmark-selection sophistication is worth its added complexity only in a small-*m* regime.

## Data Availability

The datasets presented in this study can be found in online repositories. The names of the repository/repositories and accession number(s) can be found below: https://github.com/Akramtaha98/qiawns-iomt.
