# Supplementary material for: Leakage-free evaluation of task-aware Nyström sampling for IoMT intrusion detection
Source: Front Artif Intell. 2026 Sep 28;9:1957727. doi: 10.3389/frai.2026.1957727 (PMC13655100; doi:10.3389/frai.2026.1957727)
Supplement: Supplementary file 1 [file Data_Sheet_1.pdf]

Supplementary Material S1

1 MATCHED-SEARCH CONFUSION MATRICES

The main text (Sections 4.1 and 4.2 of the primary manuscript) reports each dataset's *a priori*-configuration confusion matrix and notes that the matched-search configuration's confusion matrix is visually near-identical at print scale; both matched-search matrices are provided here in full for completeness.

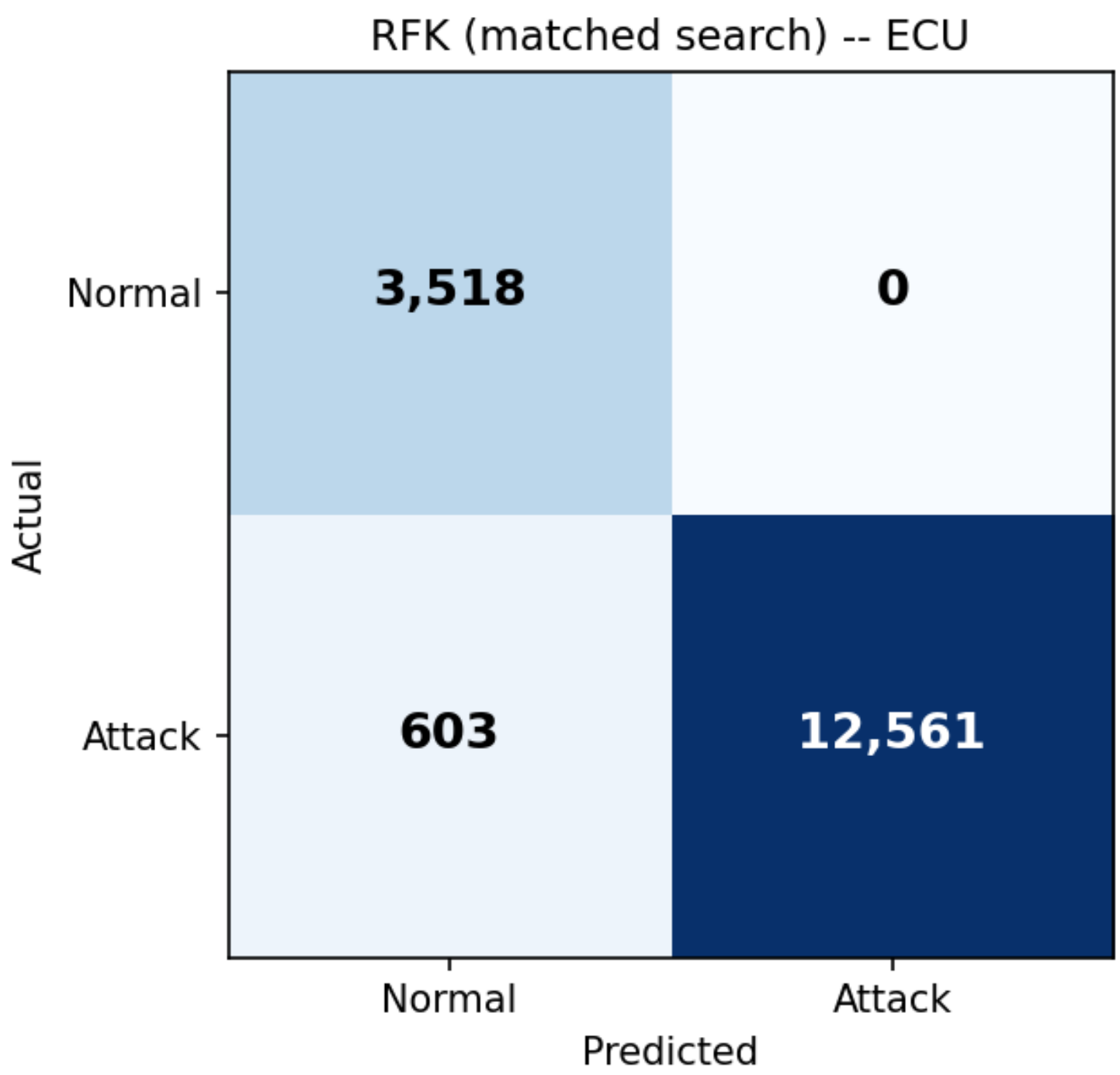

Figure S1. Confusion matrix, ECU-IoHT test set, RFK (matched-search configuration).

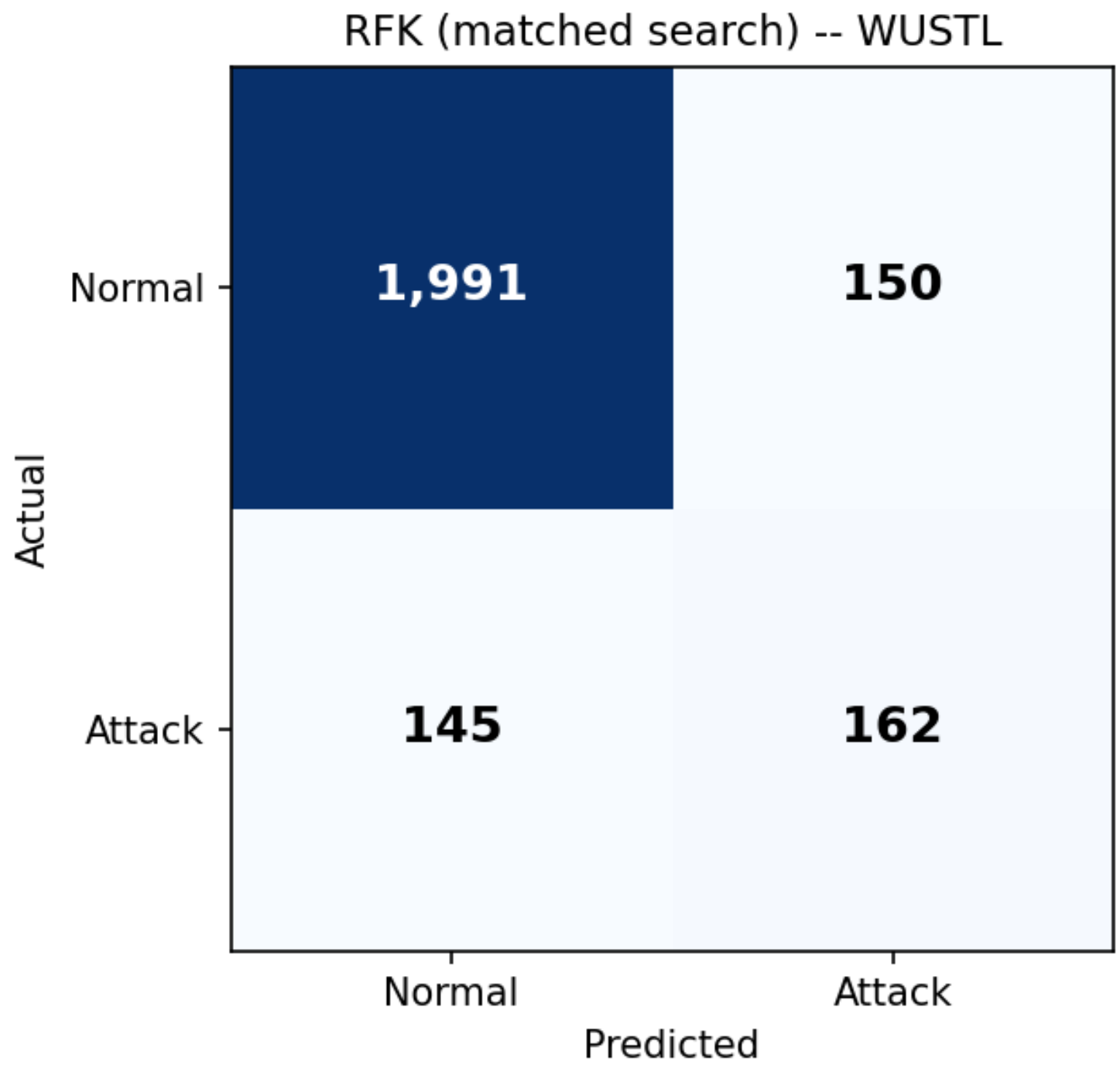

Figure S2. Confusion matrix, WUSTL-EHMS-2020 test set, RFK (matched-search configuration).
